# Experimental Insights into the Cognitive Significance of Early Stone Tools

**DOI:** 10.1371/journal.pone.0158803

**Published:** 2016-07-08

**Authors:** Mark W. Moore, Yinika Perston

**Affiliations:** Stone Tools and Cognition Hub, Archaeology, University of New England, Armidale, New South Wales, Australia; University of Oxford, UNITED KINGDOM

## Abstract

Stone-flaking technology is the most enduring evidence for the evolving cognitive abilities of our early ancestors. Flake-making was mastered by African hominins ~3.3 ma, followed by the appearance of handaxes ~1.75 ma and complex stone reduction strategies by ~1.6 ma. Handaxes are stones flaked on two opposed faces (‘bifacially’), creating a robust, sharp-edged tool, and complex reduction strategies are reflected in strategic prior flaking to prepare or ‘predetermine’ the nature of a later flake removal that served as a tool blank. These technologies are interpreted as major milestones in hominin evolution that reflect the development of higher-order cognitive abilities, and the presence and nature of these technologies are used to track movements of early hominin species or ‘cultures’ in the archaeological record. However, the warranting argument that certain variations in stone tool morphologies are caused by differences in cognitive abilities relies on analogy with technical replications by skilled modern stoneworkers, and this raises the possibility that researchers are projecting modern approaches to technical problems onto our non-modern hominin ancestors. Here we present the results of novel experiments that randomise flake removal and disrupt the modern stoneworker’s inclination to use higher-order reasoning to guide the stone reduction process. Although our protocols prevented goal-directed replication of stone tool types, the experimental assemblage is morphologically standardised and includes handaxe-like ‘protobifaces’ and cores with apparently ‘predetermined’ flake removals. This shows that the geometrical constraints of fracture mechanics can give rise to what appear to be highly-designed stoneworking products and techniques when multiple flakes are removed randomly from a stone core.

## Introduction

If the archaeological traces could have been generated by simpler actions, or if the actions could have been organized by a simpler cognitive system, then the simpler explanation must be favoured. ([1]:S12)

The persistence of stone tool-making across sequential hominin species suggests that the hominin phenotype evolved alongside this technology [2, 3] and changes in toolmaking through time and across species is evidence of cognitive evolution [4–8]. In one approach to studying cognitive abilities, tool categories—the ‘types’ archaeologists identify for comparative analysis—are assumed to reflect the forms that were intentionally produced by hominin toolmakers [9, 10]. Cognitive ability is inferred from the regularity of geometrical attributes of artefact types because metrical conformity suggests greater attention to design and more advanced cognition [8, 11–17]. In a second approach, the technical complexities of tool manufacture—a relatively direct reflection of cognitive ability—are assessed through stone-flaking experiments by modern flintknappers. The experimental approach includes narrative descriptions of how stones are transformed into types [18–29]; empirical studies of brain-imaging [30–35] and body kinaesthetics [36–40]; and theoretical modeling of the complexities of knapping [41–44]. Many explanations of cognitive evolution draw on both typological and experimental analyses because regularity of geometrical attributes on tool types is assumed to arise from cognitively more demanding manufacturing protocols [24, 45–49].

‘Least-effort’ stone tools ([50]:225–226) are the technological baseline against which complexity is evaluated because they require the least amount of skill to produce [17]. Least-effort flaking is conditioned by the size, shape, and flaking quality of the raw material, and the range of tools forms in a least-effort assemblage can be largely explained by these variables combined with the contingencies of tool function, the nature of the stone, and the distance to the stone source [21, 22, 50]. More advanced cognitive performance can be inferred from assemblages where the ‘standard forms begin to be hacked out regardless of the size, shape and flaking properties of the initial raw material’ ([50]:233). The bifacial handaxe is widely accepted as early evidence of stone-flaking to produce a ‘standard form’; other examples include ‘predetermination’ by purposeful core manipulation to produce flakes [51–58], blades [59], and polyhedrons [49]. However, for reasons related to the geometries of stone-flaking, certain characteristics and attributes of these relatively ‘advanced’ tools may occur in the simplest approaches stone flaking, a phenomenon referred to as the ‘spandrel effect’ [42]. Evaluating this theoretical possibility is difficult because the elements and attributes of a least-effort baseline technology are poorly described or extrapolated from uncontrolled knapping experiments that attempted to replicate archaeological assemblages defined *a priori* as least-effort.

Here we define least-effort flaking in reference to a model of the structure of stone flaking [41, 42] and present the results of novel experiments that approximate least-effort flaking. We reverse the approach of traditional replications studies: rather than inferring cognitive performance from the experiences of a modern flintknapper replicating a typologically-defined gestalt, we instead create a simple approach to stoneworking by explicitly disrupting the hierarchical, ‘thinking ahead’ a modern flintknapper inevitably brings to the replication task [42]. In describing the experimental products, we identify progressive changes, or lack of changes, in commonly measured attributes on cores and flakes; we also describe the initial development of core morphologies and their continuity or discontinuity as least-effort flaking proceeds.

## Experimental Design and Methodology

Controlled stone-flaking involves the arrangement of irreducible behavioural ‘cells’, called ‘flake units,’ to produce effects [41, 42, 60]. A flake unit consists of the geometrical identifications and gestural actions that are necessary to strike a flake from a stone. The phenomenon that allows flakes to be removed sequentially is the simultaneous destruction and realignment of zones of ‘high mass’ on the core face with each flake removed. When a flintknapper works intentionally towards a preconceived goal, rearrangements of high mass are predicted and acted on strategically and tactically [24, 61] and flake units are arranged hierarchically [41]. Hierarchical strategies are necessary to create exceptionally complex stone tools, and are a hallmark of the cognition underlying technical skills in modern humans [62–64].

But, because reconfiguration of mass is inevitable when a flake is removed, it is possible to progressively change the form of the core with no more intention than is inherent within the flake unit itself (the intent to remove a single flake). In this more simple process, core forms are inevitably created because mass arrangements change with each flake removed, yet flake units can be linked together in simple chains without a higher-level goal of creating a ‘standard form’ [41, 42]. In this study, ‘least-effort’ flaking refers to this approach to stoneworking. An empirical problem for archaeologists studying cognitive evolution—and one that is rarely acknowledged or explored in detail (cf. [4]:178)—is differentiating the inevitable products of simple ‘least-effort’ chaining from products that could only have been created by hierarchical strategies. The know-how of modern human flintknappers is traditionally the deciding factor on whether hierarchical flake unit arrangements were necessary to achieve the analysed effect, but the propensity for modern humans to rely on hierarchical reasoning [62–64] raises the possibility that these strategies have been unnecessarily projected onto hominin stoneworkers [42].

Our experimental protocols were designed to explore least-effort flaking by explicitly disrupting hierarchical thinking and forcing an experienced flintknapper (MWM) to produce flakes in simple chains. This was accomplished by denying the knapper a choice in how to progressively reduce the core; instead, the knapper identified all potential platforms on the experimental core and a random number generator was used to choose the platform to strike. This effectively prevented the knapper’s use of strategic and tactical reasoning to reduce the core and thwarted unconscious tendencies to work towards preconceived goals or ‘standard’ core forms. The experimental core and flake assemblage was created by linking flake units in simple chains, and is one form of ‘least-effort’ stone flaking [41, 42].

Since the focus of the experiments was on the core and flake morphologies created by flake unit chains, an individual flake unit was an experimental ‘black box,’ viewed in terms of its output rather than its internal workings. The knapper was given uninhibited scope to apply his intuitive assessments and gestural know-how to remove as large a flake as possible, of whatever shape, from the selected unprepared platform using direct freehand hard-hammer percussion. The zones of high mass included rounded natural surfaces covered in cortex, angular enhanced-mass islands of flake scars and arrises, and combinations of these. This ‘maximisation protocol’ was an unambiguous goal contingent only on aspects internal to the flake unit, such as indentor selection, mass configuration on the core, flaking angle, strength of blow, hand-eye coordination, etc. The maximisation protocol may not characterise early hominin intentions—in many cases, such as retouching to create tool edges, this was evidently not the case—but this protocol was an experimental necessity to establish a clearly-defined starting point for exploring the ‘baseline’ assemblage the experiments created. The influence of the maximisation protocol on the experimental results, as well as other aspects of the experimental design that may have influenced results, are discussed in S1 Text.

The maximisation protocol affected how MWM identified and defined suitable platforms. Platform identification and numbering involved an estimate of allometric effects combined with the goal to maximize flake size. For example, if a cobble or flake blank had a knappable platform that extended continuously along a stone for 12 cm, and it was estimated that core mass was suitable for striking 6 cm-wide flakes from it, two platforms were identified (spaced appropriately); but if it was estimated that idiosyncrasies of the core mass would only allow the removal of 4 cm-wide flakes, three platforms were identified. Four cm-wide flakes might have been struck from the former platform but these would have violated the maximization protocol.

The experiments consisted of the reduction of 29 large silcrete and mudstone cobbles and 30 medium-sized silcrete flakes (Table 1) until they dropped below the arbitrary-defined target weights of 400 grams for cobbles and 25 grams for flake blanks. The stones selected for knapping were moderately tough, ranking about 4.0 to 4.5 on Callahan’s ([65]:Table 3) lithic grade scale, and similar in knapping quality to quartzite or coarse-grained chert. Each blow removed a flake and created a core with a new morphology, resulting in data for 1115 cores from the 59 stones (S1 Table). Cores were produced sequentially from each stone, but, because platform selection was randomized, reduction effectively began anew with each flake removed. The cores were classified into two typological schemes. As each flake was removed, the core and flake was classified by MWM into technological types (after [66]). Then each flake was conjoined back onto the core with double-sided tape and, as each flake was added, the core was classified by YP into Isaac’s [67] morphological typology developed for the Middle Pleistocene assemblage from Olorgesailie, Kenya. The Olorgesailie typology was chosen because it accommodates and describes the wide range of variation of core types in early stone artefact assemblages. (The results of the typological study will be described elsewhere). Core and flake attributes were measured according to methods described below.

**Table 1:** Dimensions of cobbles and flake blanks reduced in the experiments.

	Silcrete cobbles**, Mean +/- SD, N = 16	Mudstone cobbles***, Mean +/- SD, N = 13	Silcrete flake blanks****, Mean +/- SD, N = 30
Length	258.1 +/- 39.5	247.0 +/- 36.0	128.9 +/- 20.3
Width	203.2 +/- 15.2	191.0 +/- 27.8	93.7 +/- 17.9
Thickness	149.9 +/- 24.0	135.8 +/- 33.0	37.4 +/- 9.9
Grams	8919.7 +/- 2327.7	6833.3 +/- 2829.9	367.2 +/- 184.6
Length/Width*	1.27 +/- 0.2	1.30 +/- 0.2	1.41 +/- 0.3
Width/Thickness*	1.38 +/- 0.2	1.47 +/- 0.3	2.62 +/- 0.6

* Ratios presented here and elsewhere were calculated from raw data rather than summary data.

** The fully-rounded silcrete cobbles were from gravel bars of the Hunter River, New South Wales. This fine- to medium-grained silcrete is a composed of small- (<0.25 mm) to medium-sized (0.25–0.5 mm) sub-rounded quartz grains in a silica matrix and is similar in appearance to orthoquartzite [68]. The outer surfaces of the cobbles were pocked with shallow incipient cones from water-rolling.

*** The fully-rounded indurated mudstone cobbles were also from the Hunter River, and consisted of metamorphosed silt/clay or volcanic ash [69]. The stone is slightly rough or chalky in appearance and insufficiently silicified to develop incipient cone cortex.

**** Flake blanks struck from angular blocks of pedogenic silcrete from a modern gravel quarry at Armidale, New South Wales. The stone consists of medium-sized (0.25–5.0 mm) angular chert fragments distributed irregularly in a silica matrix. Fossil rootlets up to 2.0 mm in diameter are common and the degree of silicification varied between and within individual stones.

Stone hammers were not used in the experiments because the heavy blows necessary to strike flakes from large stone cobbles causes significant hammerstone attrition after a few blows and, eventually, catastrophic failure. To maintain indentor consistency over the more than 1000 percussion blows delivered in the experiments, and to aid in replication of these experiments, five solid copper bars were used (Table 2). These indentors retained their original mass across all of the experiments. Copper indentors deformed slightly on impact and were therefore somewhat ‘softer’ than stone hammers, and in 33 cases it proved necessary to use a non-deforming steel hammer to initiate fracture. Indentor size was scaled to the size of the intended flake, with the larger indentors used mostly in cobble reduction and smaller indentors in flake blank reduction. The copper indentors were used in the same manner as stone hammers, held either in a precision grip with the tip aimed at the core platform or in a power grip and swung like a club or billet. The knapper supported large cores on cloth padding on the laboratory floor, and, once it became smaller than, on average, 1752.1 grams (starting at about blow 13), the core was held in the non-dominant hand. To better simulate a ‘natural’ knapping posture (e.g., [70, 71]), flaking was conducted with the knapper on his knees or sitting on a low seat (19 cm high). Cores were not supported on or braced by the leg in the manner of Brandon gunflint knappers (see [72]), as commonly practised by modern flintknappers (cf. [40]).

**Table 2:** Sizes of indentors and frequency of use.

Indentor*	Length (mm)	Diameter (mm)	Weight (g)	Blows, cobble reduction, N (%)	Blows, flake blank reduction, N (%)
1	117.31	59.07	2674	215 (34.1)	0
2	119.23	49.40	1871	175 (27.8)	1 (0.2)
3	125.24	38.15	1218	134 (21.3)	15 (3.1)
4	102.82	31.80	718	72 (11.4)	184 (37.9)
5	100.61	22.21	335	5 (0.8)	285 (58.8)
6 (steel)	98.74	44.40	1265	29 (4.6)	0

*1–5: solid copper bars. 6: steel hammer head. The masses of indentors 1–3 are ‘very heavy’, ‘heavy’ and ‘medium’ [73].

A ‘blow’ is defined here as a strike that removed a flake. Multiple strikes were often necessary to induce fracture, particularly in initial cobble reduction. Flakes sometimes split in a siret fracture ([74]:34, [156]) or suffered platform collapse. In these cases, the fragments were reassembled and treated as a complete flake. Quantities of ‘shatter’ were produced during reduction, consisting of spin-off flakes such as eraillures, lances, and detached finials, as well as small angular pieces and cortex fragments. Shatter measuring greater than 5 mm was weighed after each blow, and shatter and dust smaller than 5 mm was weighed together at the end of core reduction. Small flakes were sometimes produced through ‘spontaneous retouch’ caused by the lateral or distal margins of a flake levering against the core at the moment of flake detachment, particularly when striking very large flakes using the padded laboratory floor to support the core. Spontaneous retouching flakes were weighed with the shatter.

Stoneworking is a sport-like activity that integrates human movement with objects. As such, it is prone to variation in result because of human mis-timing and the vagaries of the objects themselves (such as inconsistencies in the stone). The possibility of these variations was exacerbated by the experimental design because data recording prevented a natural flaking ‘rhythm’ to develop. To gauge the effects of human error and material factors in this study, the knapper was required to predict blow outcomes in terms of flake size and platform quality. To predict flake size, the knapper drew onto the core the outline of the largest flake that might be struck from the selected platform (cf. [75]); this outline became the knapper’s goal. The outline included the predicted plan shape and boundary of the flake’s platform. Platform quality was defined as the likelihood that the goal would be achieved given the nature of the material and platform/mass geometry. The knapper scored the platform configuration on a five-point scale, with 3 deemed an average chance of success, 5 the maximum chance, and 1 a minimal chance. After the flake was removed, the knapper scored ‘blow success’ relative to the outline using a five-point scale, with 3 as meeting the prediction, 5 as substantially exceeding it, and 1 as failure (because of platform collapse or mis-hits). Table 3 shows that most platforms and blow success were ranked 3, although on average the actual results were assessed higher than predicted results.

**Table 3:** Platform count per core and platform quality/blow success.

	Cobbles, Mean +/- SD	Flake Blanks, Mean +/- SD)
Platform quality*	2.48 +/- 0.83	2.53 +/- 0.70
Blow success**	3.04 +/- 1.16	3.21 +/- 0.99

*Assessed by the knapper prior to striking the flake.

**Assessed by the knapper after striking the flake.

The accuracy of the knapper’s predictions were evaluated empirically by measuring the distance between the outside of the predicted outline and the edge of the flake or flake scar at four points: one on each lateral edge at the midpoint of the flake/scar, one at the distal edge, and one at the point of force initiation. When the knapper under-predicted the result, the flake undercut the line and it was preserved on the flake’s dorsal surface. When the knapper over-predicted the result, the line remained on the core. Lateral edge predictions were the most accurate (Table 4) and despite very different core sizes between the cobble and flake blank experiments, flakes were predicted within a consistent 13% margin of error. Distal edge predictions were less accurate because of unpredicted overstrikes. Platform depth estimates were the most inaccurate because of an unconscious, and perhaps idiosyncratic, gesture on the part of the knapper: in cases when the first blow failed to initiate a crack, the second blow was delivered slightly closer to the platform edge. This shallower strike decreased the size of the potential flake by placing the crack path closer to the core face, with the result that the crack could be successfully initiated without increasing the strength of the blow.

**Table 4:** Difference between predicted flake outlines and actual flake boundaries for successful flake removals.

	Cobble		Flake Blank	
	Mean Difference in mm	Difference as % of flake attribute	Mean Difference in mm	Difference as % of flake attribute
Lateral margin	11.41 (N = 636)	12.53 * (N = 636)	4.76 (N = 681)	12.73 * (N = 681)
Distal margin	16.47 (N = 315)	16.75 ** (N = 315)	7.40 (N = 343)	21.94 ** (N = 343)
Platform depth	6.8 (N = 310)	32.27 *** (N = 305)	3.98 (N = 344)	40.45 *** (N = 333)

* Percent of corresponding flake’s maximum width.

** Percent of corresponding flake maximum length.

*** Percent of corresponding flake’s platform depth.

## Technology of Cores and Flakes

### Core attrition

**Fig 1:**
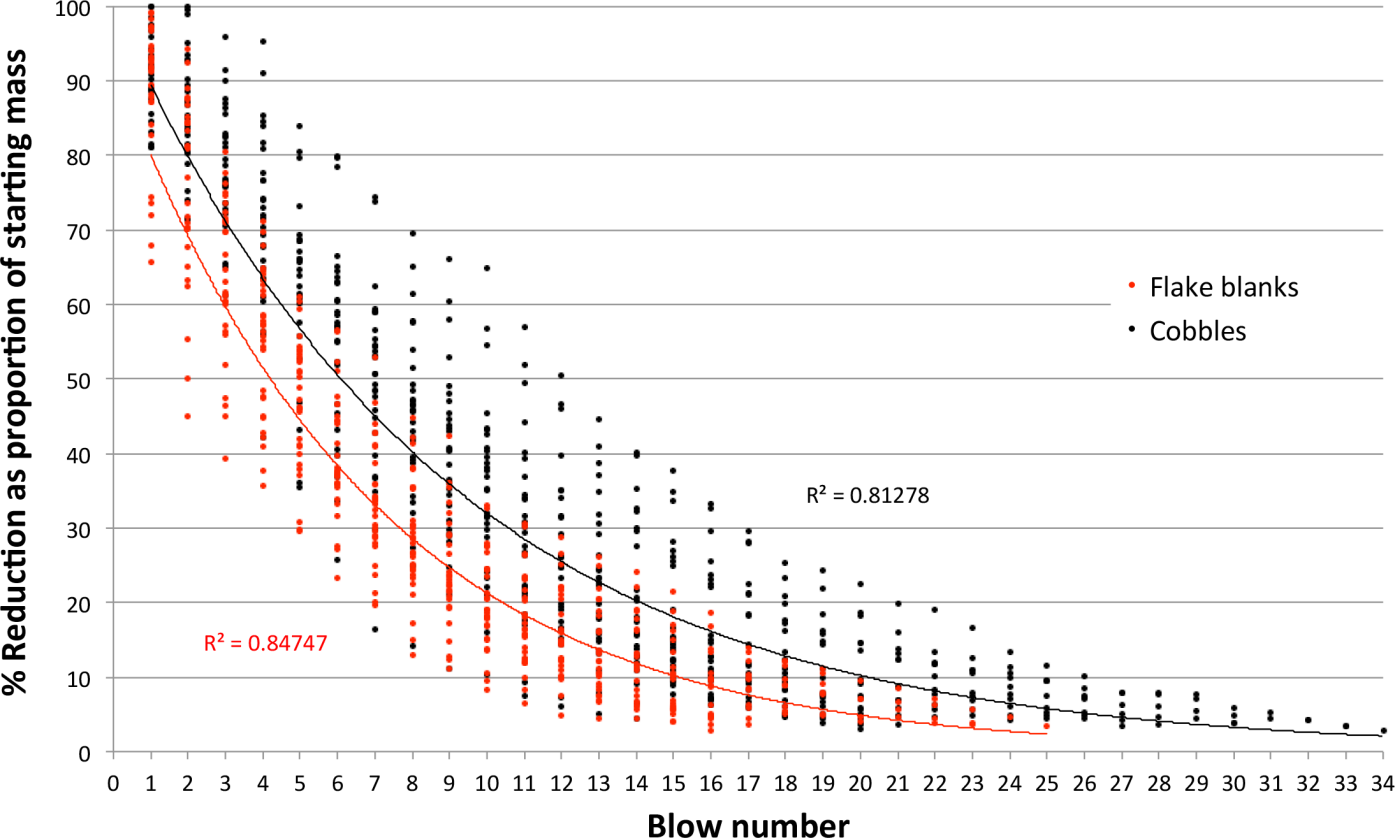
Scatterplot showing the decrease in total core mass relative to reduction interval (blow number).

**Fig 2:**
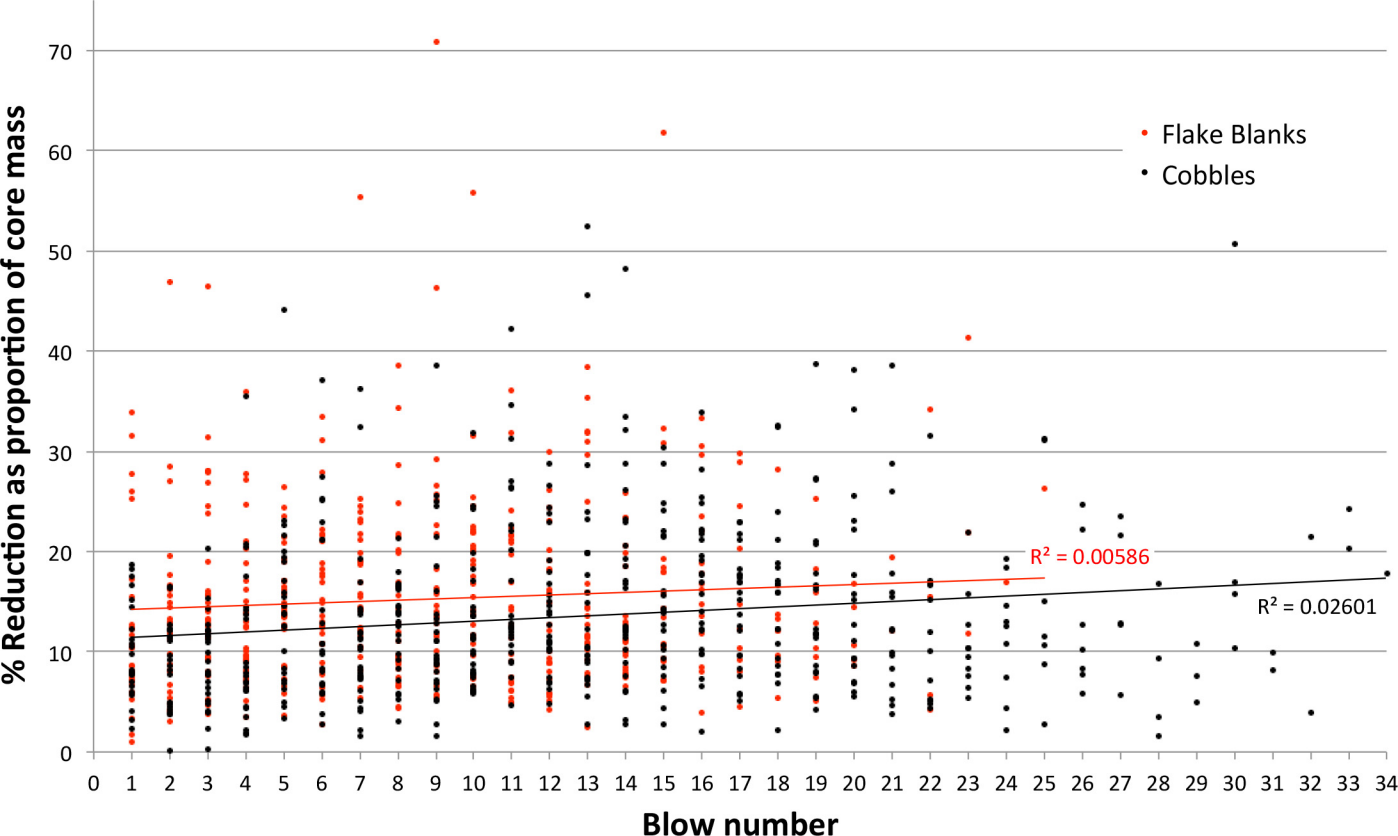
Scatterplot showing the decrease in individual core mass caused by the removal of a flake, relative to reduction interval (blow number).

Cores were weighed after each flake removal and no flakes were struck after a core dropped below the experimental target weight (Table 5). Reduction decreased the initial starting mass by about 92–95% through, on average, 16 to 22 blows. Core reduction was a continuous process with each blow composing a discrete ‘reduction interval’. The decrease in core mass, as a proportion of the starting weight, was exponential and strongly correlated with an increase in reduction interval for both cobbles (r^2^ = 0.81278) and flake blanks (r^2^ = 0.84747) (Fig 1). This is unsurprising because, as a core shrinks, the absolute size of flakes that can be struck from it declines, and flakes inevitably become an ever-decreasing proportion of the stone’s starting mass. However, when flake weight is plotted as a proportion of the core mass just prior to the blow, the strong correlation with reduction interval disappears for both cobbles (r^2^ = 0.02601) and flake blanks (r^2^ = 0.00586) (Fig 2). Although absolute flake sizes decreased with reduction, a flake’s mass relative to the core from which it was struck averaged 13.6 +/- 8.99% (N = 1103), reflecting the experimental protocol to maximize flake size. Despite this, blows struck on flake blanks removed significantly larger proportions of core mass than blows struck on cobbles (*p*<0.0001, *t* = 4.2276, df = 1101, SE 0.542). Also, significantly fewer blows were necessary to reach the target size for flake blanks (*p*<0.0001, *t* = 4.3021, df = 57, SE 1.278). The average mass of the flake blanks chosen for the reduction experiments was similar to the average mass of cobble cores at the end of reduction (Tables 1 and 5), and the greater efficiency seen in flake blank reduction is likely a previously unrecognised allometric effect related to core size rather than blank type (cf. [76, 77]).

**Table 5:** Summary data on core attrition.

	Cobbles, Mean +/- SD	Flake Blanks, Mean +/- SD
Starting grams	7984.4 +/- 2729.4 (N = 29)	367.2 +/- 184.6 (N = 30)
Target grams	400	25
Finished core grams	364.0 +/- 48.5	21.7 +/- 3.1
Percent decrease in mass	94.8 +/- 2.1%	92.4 +/- 4.2%
Number of blows to achieve target	21.7 +/- 5.7	16.2 +/- 4.0
Percent of core mass removed per blow	12.59 +/- 8.65% (N = 630)	14.88 +/- 9.26% (N = 485)

### Platform counts and persistence

**Fig 3:**
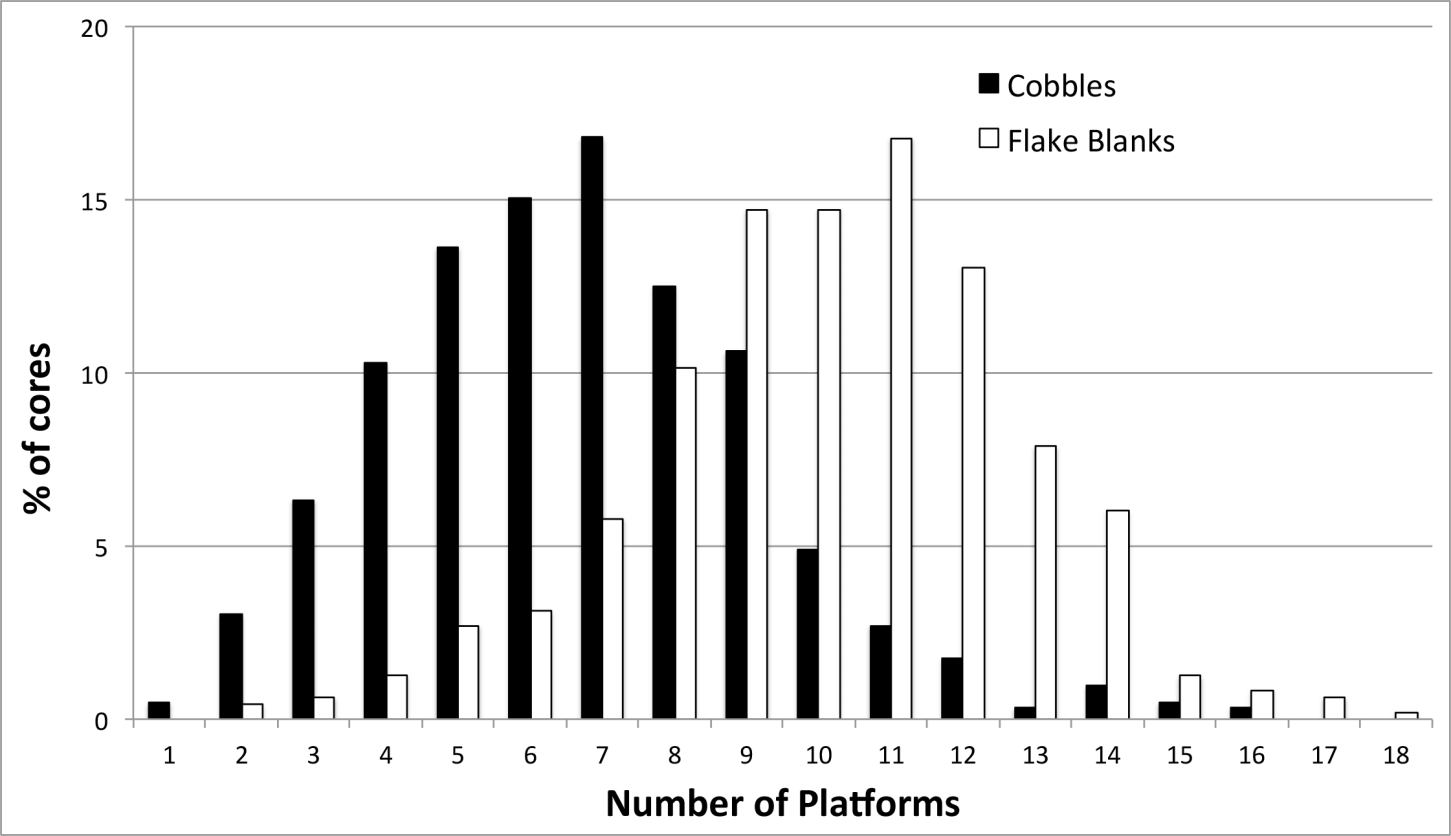
Comparison of platform counts on cores made on cobbles and flake blanks.

Some 3338 platforms were identified across the 59 reduction experiments and 32.2% of these were chosen by the random number generator and removed (Table 6). The remaining 67.8% of platforms were either eliminated by undercutting (55.4%) or were retained on the core at the end of the experiment (12.4%). Platforms sometimes survived up to 20 blows, although most were eliminated after 3 blows. These figures vary between flake blanks and cobbles because, on average, flake blank cores tended to have about 4 more identified platforms than cobble cores (Fig 3). Because a platform on a flake blank was slightly less likely to be randomly selected, it was also less likely to be eliminated by striking. Conversely, more potential platforms were concentrated on flake blank cores and a greater number was likely to be eliminated by undercutting.

**Table 6:** Platforms identified and eliminated.

	Cobbles		Flake Blanks	
	N	%	N	%
Total number of identified platforms	1637		1740	
Platforms eliminated by striking	630	38.5	485	27.9
Platforms eliminated by undercutting	797	48.7	1051	60.4
Platforms not eliminated	210	12.8	204	11.7
Platform persistence (median number of blows a platform remained viable)*	3		3	

*Non-eliminated platforms removed from the total in this calculation.

A greater number of potential platforms were identified on flake blank cores (10.14 +/- 2.58) than on cobble cores (6.66 +/- 2.54). This can be attributed to the angular shape of flake blanks at the outset of reduction and the acute angle between the ventral and dorsal surfaces. Suitable platforms were identified on flake blank ventral surfaces with slightly greater frequency (52.8%) than on dorsal surfaces (47.2%). The propensity for flakes in the archaeological record to be retouched from ventral platforms may be because the regularity of the ventral surface allows for contiguous blows, rather than the nature of the platform edge-angle.

### Platform creation and elimination

**Fig 4:**
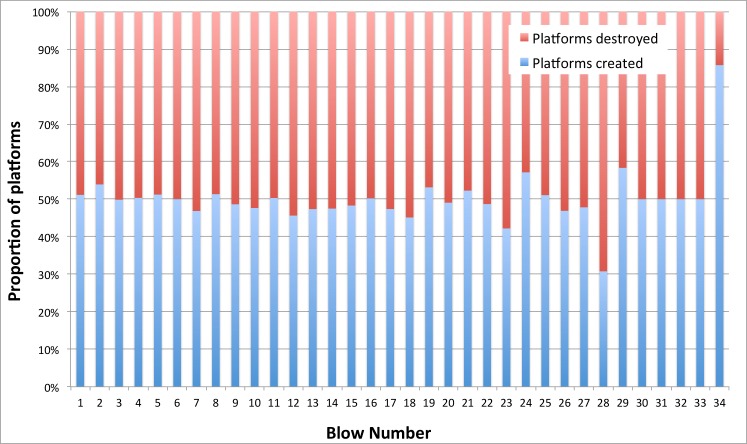
Proportion of platforms created and destroyed by reduction interval (blow number) for cobble and flake blank reduction.

The total number of platforms created and eliminated did not differ significantly between cobble and flake reduction (*p* = 0.3170, *t* = 1.0010, df = 2228, SE = 0.061). Platforms were created and eliminated at about the same rate through the reduction process in both sets of experiments (Fig 4). Although one platform was always eliminated by flake removal, and others were eliminated by undercutting, this was balanced by the addition of new platforms through changes in geometrical configurations. In cobble reduction, two platforms tended to be created and eliminated on average with each blow. Flake blank reduction proved more dynamic, with on average 3 platforms created and eliminated with each blow (Table 7). In some experiments, considerably greater numbers of platforms were eliminated than created (or visa-versa), but this difference never exceeded 5 platforms for cobbles or flake blanks. On other occasions, platforms were not created, or a blow only eliminated the struck platform. Platform stasis like this was more common in cobble reduction than in flake blank reduction (Table 8), again indicating that more dynamic platform transformations occurred in reducing flake blanks. This is likely related to the allometric effects discussed previously, where greater proportions of core mass were removed per blow on smaller flake blank cores than on larger cobble cores—in turn resulting in greater platform elimination and creation per blow.

**Table 7:** Platform creation and elimination per blow.

	Cobbles (N = 630 blows)			Flake Blanks (N = 485 blows)		
	Platforms created per blow	Platforms eliminated per blow	Difference per blow*	Platforms created per blow	Platforms eliminated per blow	Difference per blow*
Mean +/-SD	2.27 +/- 1.46	2.21 +/- 1.25	1.08 +/- 1.01	2.92 +/- 1.38	3.13 +/- 1.45	1.20 +/- 1.01
Median	2	2	1	3	3	1
Maximum	8	9	5	8	8	5
Minimum	0	1	0	0	1	0

* Difference between the number of platforms created and eliminated, calculated after each blow.

**Table 8:** Number of instances when a blow failed to create new platforms or eliminate pre-existing platforms.

	Cobbles (N = 630 blows)	Flake Blanks (N = 485 blows)
New platforms were not created by flake removal	50 (7.9%)	10 (2.1%)
Pre-existing platforms were not eliminated by flake removal*	214 (34.0%)	50 (10.3%)

*Not including the platform that was struck to remove the flake.

### Platform locations

**Fig 5:**
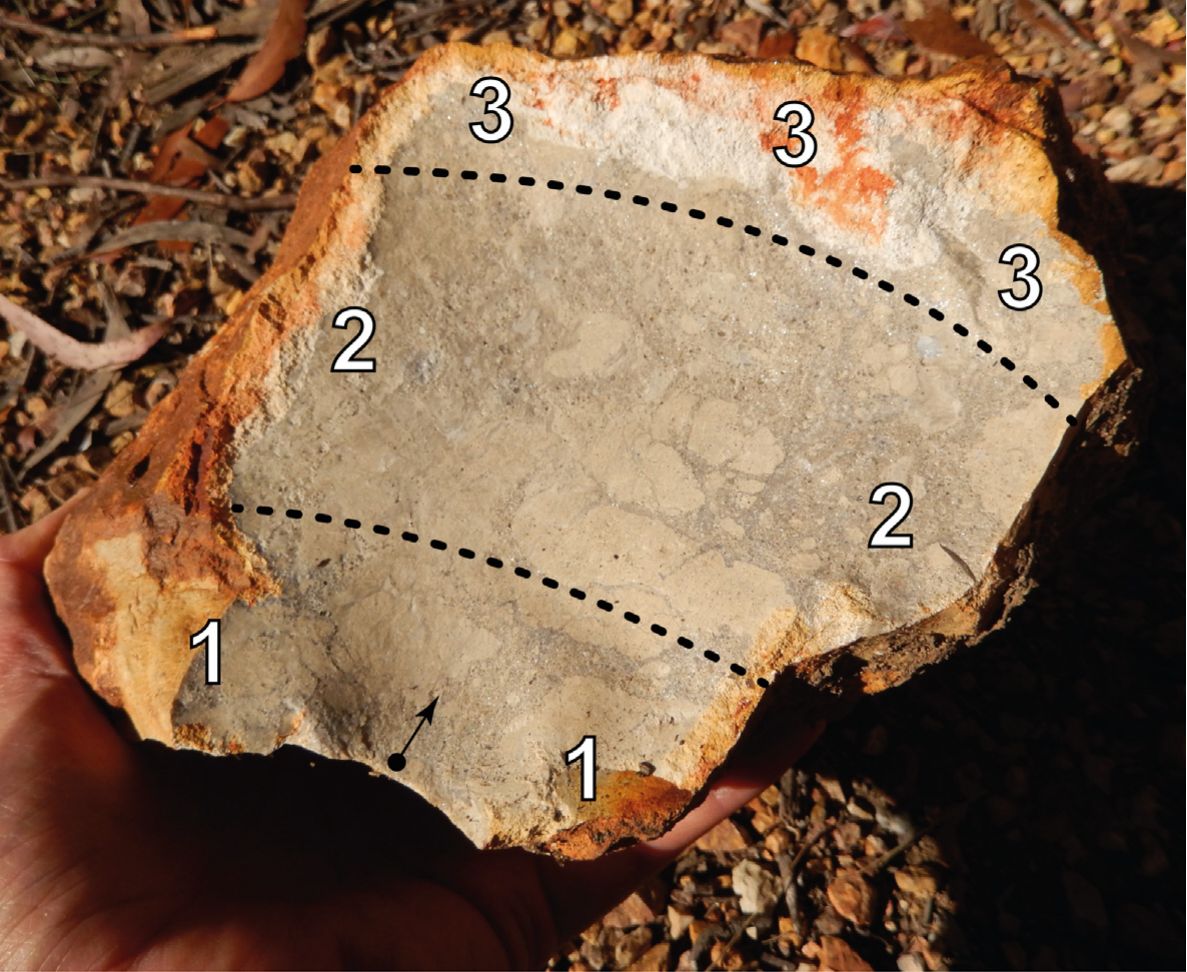
Negative scar on a silcrete cobble showing platform positions. ‘Position 1’ platforms are located in a zone extending from the proximal end to 30% of the scar’s length, ‘Position 2’ are located between 30% to 80% of the scar’s length, and ‘Position 3’ platforms are located on the scar’s distal end, beyond 80% of its length. Negative scars are always concave and many of the scars produced in the experiments were markedly so. Because of this factor, and the difficulty of landing percussion blows in a concave surface, platforms were usually located within the 10% of the scar’s width extending from the scar’s edge. ‘Position 4’ platforms are cortical surfaces; ventral surfaces or dorsal scar remnants from core reduction on dorsal surfaces of flake blank cores; or small core scar remnants that could not be classified into the other platform types.

The locations of struck platforms were classified into four possible positions, as shown in Fig 5. The frequency of the positions on the experimental cores are presented in Table 9. Since the platforms for the struck flakes were selected randomly, the Table 9 data is also a proxy for the overall distribution of potential platforms on the experimental blanks.

**Table 9:** Positions of platforms randomly selected for striking.

	Cobbles*		Flake Blanks	
	N	%	N	%
Position 1	184	34.0	165	34.0
Position 2	78	14.4	49	10.1
Position 3	70	12.9	39	8.0
Position 4, cortical surface	89	16.4	20**	4.1
Position 4, non-cortical surface	121	22.3	212	43.7
Total	542		485	

* Not recorded for cobble experiments 1–4.

** A cortical platform was created in the flake blank reduction experiments when a blow was delivered to a dorsal cortical facet. Unlike cobble cores, which were 100% covered by cortex at the outset of reduction, the amount of dorsal cortex coverage varied between flake blanks, and as a result the proportion of cortical platforms selected in flake blank reduction is not considered meaningful.

**Fig 6:**
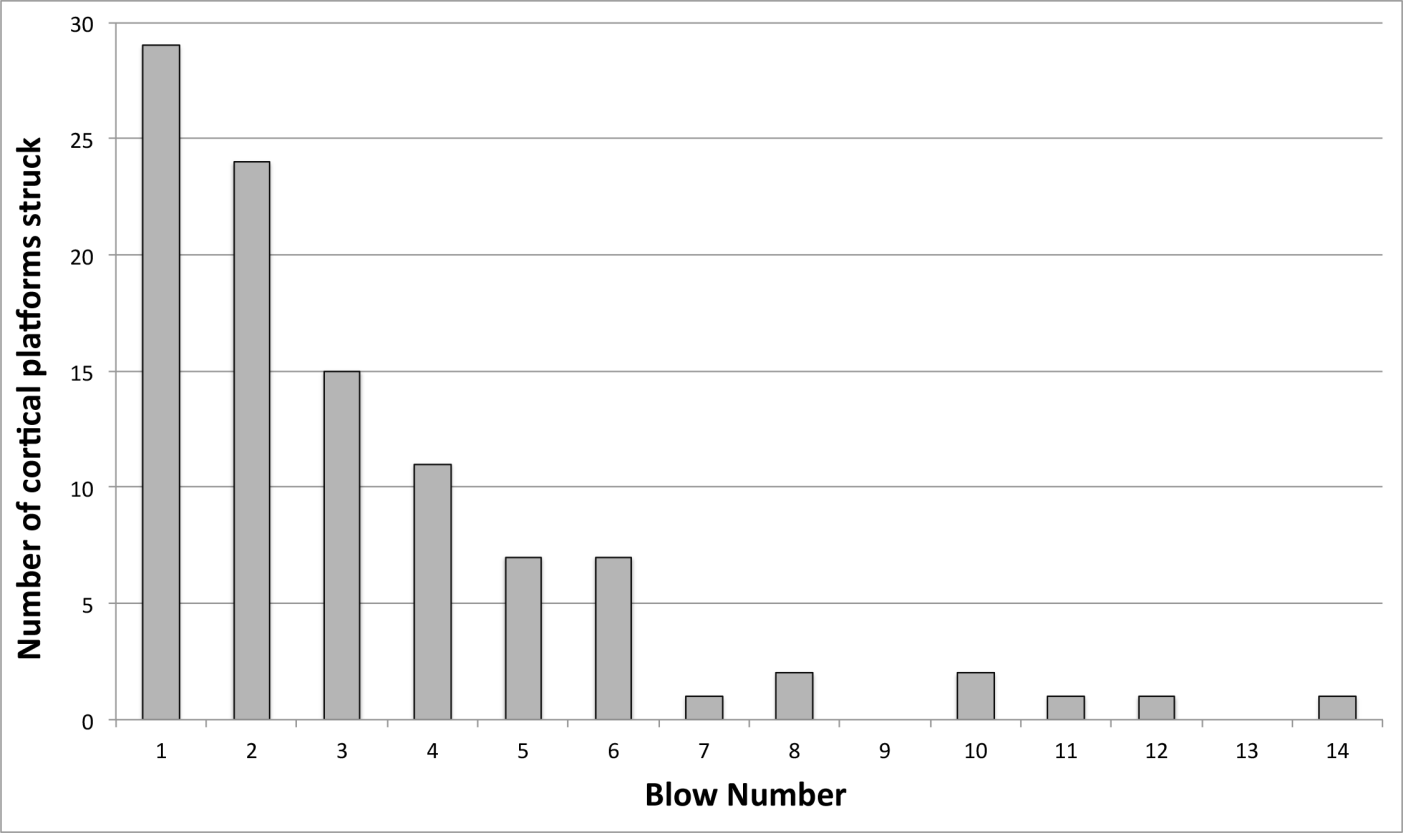
Frequency of cortical platforms by reduction interval (blow number).

In the cobble reduction experiments, the first blow was always onto a Position 4 cortical platform. As reduction proceeded, potential platforms on cortical surfaces decreased in number without replacement, while suitable platforms on flake scars proliferated (positions 1 to 3). Most strikes onto cortical platforms occurred within the first 3 blows and they rarely occurred after 6 blows (Fig 6). Despite the fact that the starting cobbles were entirely covered in cortex, only 16.4% of the struck platforms in the cobble experiments were on cortical surfaces.

Over half of the platforms in both sets of experiments were located on negative scars (positions 1–3), and of these, most were in Position 1, close to the edge of the core and the proximal end of the flake scar (Table 10). This location near the core edge allows the most ready access to core mass on the face adjacent to the platform. On cobbles, proportionately greater numbers of negative-scar platforms were located elsewhere (positions 2 and 3), reflecting more diverse reduction opportunities on globular stones than on relatively flat flakes.

**Table 10:** Struck platforms on negative flake scars.

	Cobbles*		Flake Blanks	
	N	%	N	%
Position 1	184	55.4	165	65.2
Position 2	78	23.5	49	19.4
Position 3	70	21.1	39	15.4
Total	332		253	

* Not recorded for cobble experiments 1–4.

### Flake scar erasure

Scars from the earliest flake removals are progressively undercut and eliminated, or ‘erased’ [78], as reduction proceeds. Table 11 confirms that the final cores in our experiments disproportionately preserve flake scars produced later in the reduction process, although the earliest scars were often preserved on the final cores (cf. [79]). Braun et al. [78] experimentally explored rates of scar erasure in the reduction of Oldowan-like cores and found that flake scars were erased more quickly from small cores (<1.0 kg) than from large ones (>2.0 kg). Our experimental results demonstrate that the likelihood of scar survival is strongly correlated with reduction interval for both large cobble cores (r^2^ = 0.96) and small flake blank cores (r^2^ = 0.90), but, in contrast to Braun et al.’s [78] results, the rates of scar erasure shown by the regression lines do not differ significantly (ANCOVA F[1, 17] = 0.84, *p* = 0.372). On average, each 10% increase in reduction interval removed 2.23 +/- 1.54% (N = 314) of the scars on cobble cores, and 2.25 +/- 2.66% (N = 267) of the scars on flake blank cores. Also, the ratio of total reduction blows relative to scars on final cores for the large cobble experiments (2.01 +/- 0.46, N = 29) and the flake blanks experiments (1.82 +/- 0.32, N = 30) do not differ significantly (*p* = 0.0699, t = 1.8470, df = 57, SE = 0.103).

**Table 11:** Scar preservation on final cores, by reduction interval.

Reduction interval (% of core reduction*)	Percentage of final cores with scars preserved from the reduction interval	
	Cobbles (N = 29)	Flake Blanks (N = 30)
0–10	20.7	16.7
11–20	27.6	50.0
21–30	44.8	33.3
31–40	55.2	53.3
41–50	62.1	56.7
51–60	82.8	80.0
61–70	82.8	83.3
71–80	100.0	80.0
81–90	100.0	90.0
91–100	100.0	100.0

* Percent reduction was calculated for each blow based on the total number of blows required to reduce the core. This was collated into bins to facilitate comparison across reduction events with disparate numbers of blows.

These results show that, in our experiments, flakes were eliminated at about the same rate from large and small cores, consistent with the maximisation protocol and the allometric effects discussed previously. Flake size was directly related to the size of the core, and erasure rate was relatively uniform and consistent. In contrast, Braun et al.’s experimental goal was the removal of ‘usable flakes’ ([78]:526, cf. [80, 81]) from cobbles of various sizes, rather than flakes of maximum size. ‘Usable’, in this case, may reflect the removal of flakes of similar size from large and small cobbles; given that flakes of standard size would represent a greater proportion of small core mass than large core mass, this might explain why Braun et al.’s [78] rate of flake scar erasure was greater during the reduction of small cores.

### Inevitability of bifacial flaking and bifacial flake scar organisation

A bifacial platform edge is flaked to two adjacent core faces, whereas a unifacial edge is flaked to one face only. By this definition, a bifacial platform could not transition to a unifacial platform. Bifacial reduction occurred in all 59 reduction experiments, and bifacial edges were created when flakes were struck from platforms at the edges of negative scars created by prior flake removals, particularly in positions 1–2. Platforms in these positions proliferated as new negative scars were produced, increasing the likelihood of their random selection. Bifacial reduction was not an outcome of the knapper’s intent to create a bifacial edge, but was instead an inevitable outcome of identifying potential platforms on surfaces of negative scars created by prior flake removals.

**Fig 7:**
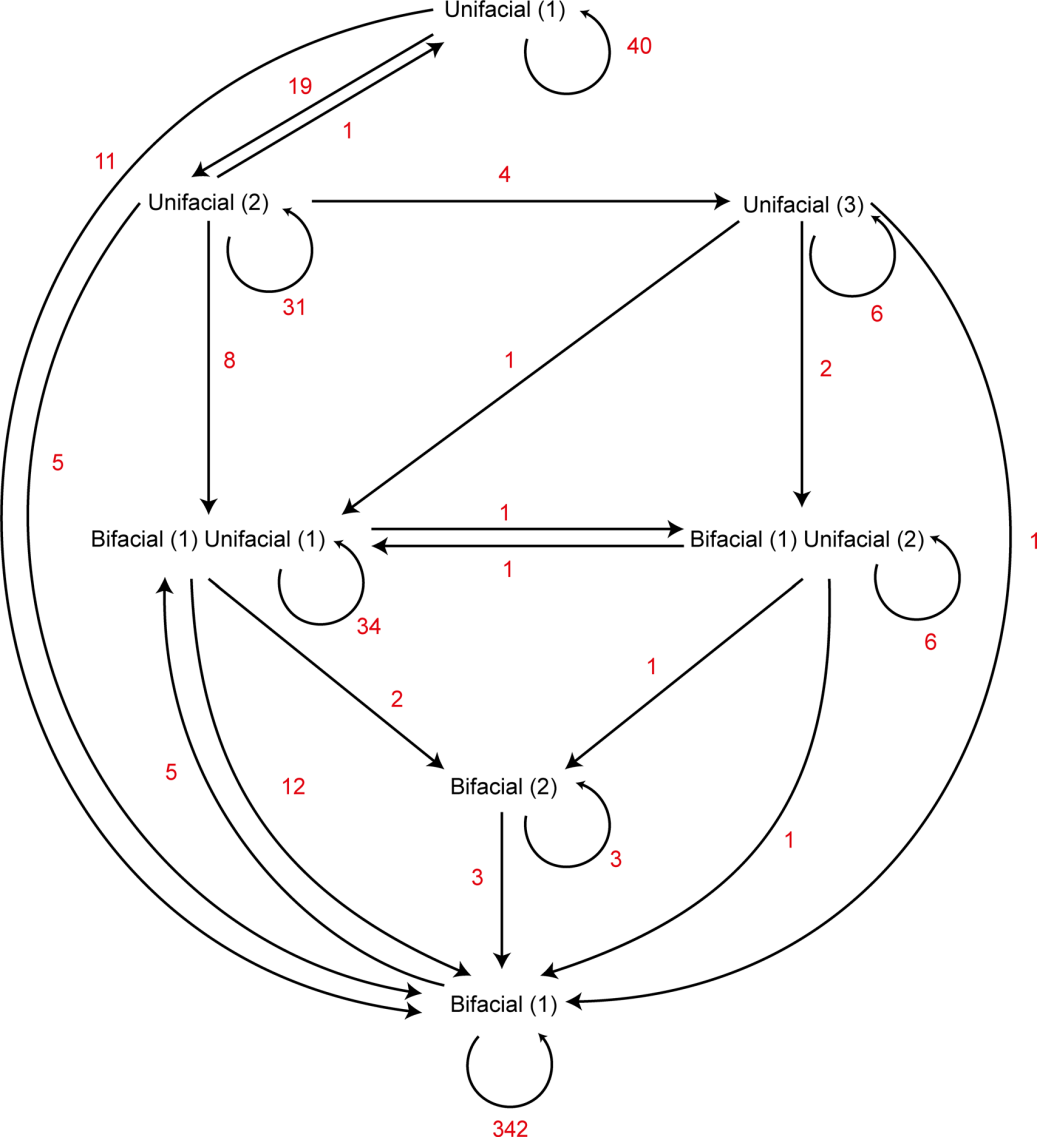
Frequency and directions of pathways in the development of bifacial edges on cores made on flake blanks. Arrows indicate the direction of the transition, and red numerals indicate the number of times a transition occurred in the experiments. The number of independent platforms are in parentheses.

Unlike globular cobbles, a flake blank consists of two volumes [82]—the dorsal and ventral surfaces—clearly defined by the blank’s edge. This separation allowed a detailed examination of the transition from unifacial to bifacial reduction in the flake blank experiments. All core reductions begin with unifacial reduction (one flake struck to one core face). Fig 7 shows the frequency of various pathways from this unifacial edge, through combinations of independent unifacial or bifacial platforms, to one bifacial platform edge. Independent platforms merged when they extended laterally and overlapped. The most common progression involved the initial production of two independent unifacial platform edges (and never more than three) followed by one of these edges becoming bifacial; subsequent reduction merged them into one bifacial platform edge. The trend was for platform complexity early in reduction quickly resolving to one bifacial edge; this occurred within 6 blows from the start of reduction in 75% of the flake blank experiments, and never required more than 12 blows.

**Fig 8:**
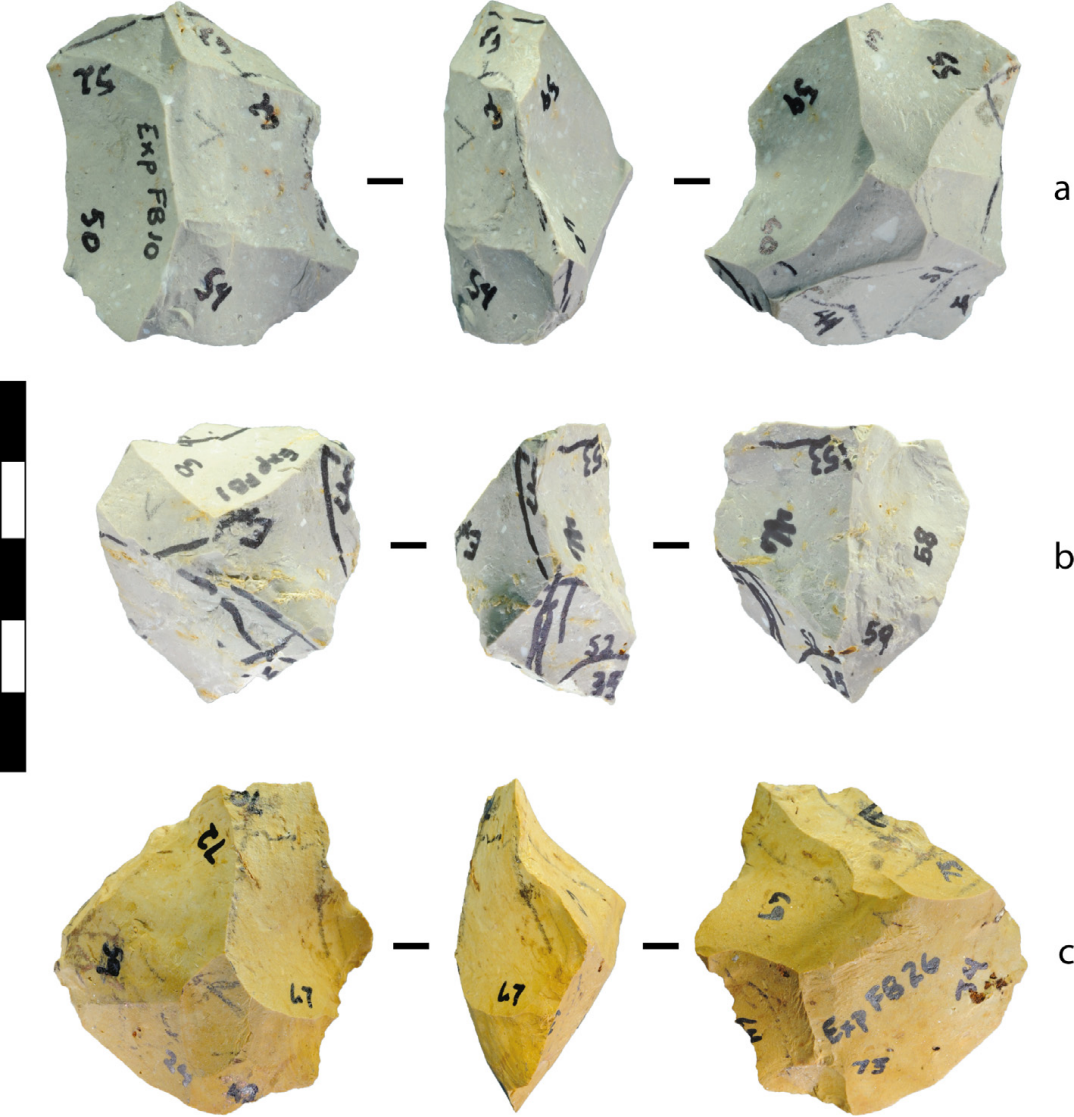
Experimental bifacial cores produced from flake blanks and displaying approximate radial symmetry. The numbers written on the cores denote potential (but unselected) platforms. (A) Silcrete, experiment 10, after 19 blows. (B) Silcrete, experiment 1, after 22 blows. (C) Silcrete, experiment 26, after 23 blows. Scale 50 mm.

**Fig 9:**
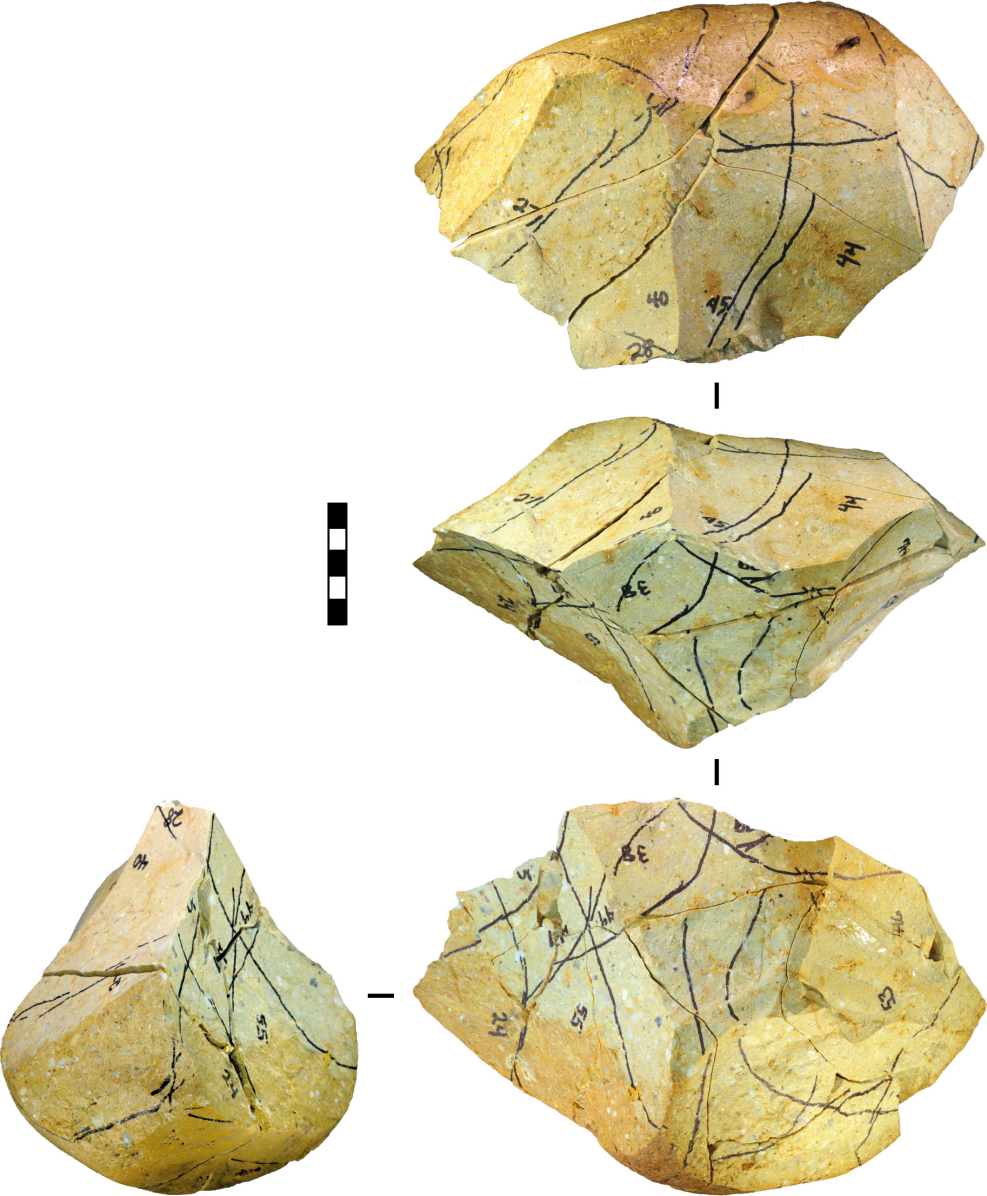
Experimental cobble core displaying a ‘chopper’ morphology. Silcrete, experiment 17, after 17 blows. The rounded morphology on the side of the cobble prevented the identification of potential platforms in that location, and reduction up to this point in the experiment progressed bifacially towards this rounded surface. Scale 50 mm.

**Fig 10:**
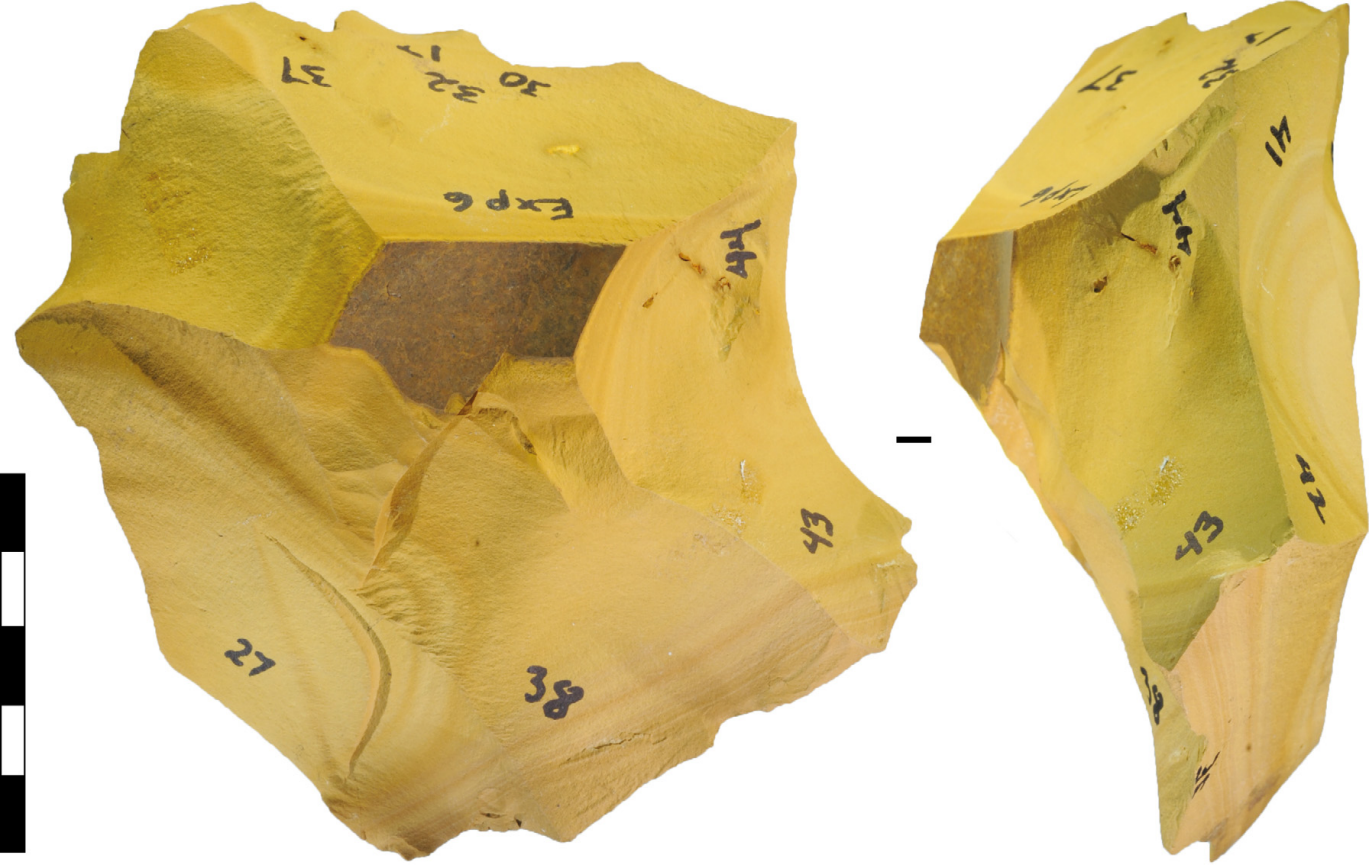
Experimental bifacial core produced from a cobble displaying approximate radial symmetry. Mudstone, experiment 6, after 13 blows. Scale 50 mm.

Initial platforms on flake blanks were distributed around the perimeter edge that defined the two core volumes. This organisation strongly influenced the development of a bifacial edge, leading to centripetal (‘radial’) scar patterns, with flakes struck from the bifacial perimeter towards the centre (Fig 8). Radial patterning was produced without a deliberate intention to do so. In contrast to flake blanks, cobbles lacked a distinct edge and the distribution of potential platforms at the outset of the experiments tended to be more variable. Also, significantly fewer starting platforms were identified on the unmodified cobbles (6.38 +/- 2.27) than on the unmodified flake blanks (10.40 +/- 1.52) (*p*<0.0001, *t* = 8.018, df = 57, SE = 0.501). The removal of the first flake from a cobble (a ‘cobble-opening’ or entame flake ([83]:339–342) usually created two new platforms, and often more than two, on the negative scar. This proliferation increased the likelihood that a negative-scar platform would be randomly chosen rather than a platform on a cortical surface, an effect that tended to increase with each flake removed. The effect on some cobble cores was the creation of a bifacial edge around only part of the cobble’s periphery, and the repetitive striking of flakes from this bifacial edge as reduction proceeded. Core ‘choppers’—with a rounded cortical surface opposite a bifacially flaked edge—were unintentionally created by this process (Fig 9), although this is sometimes considered an intentional feature of tool design (e.g., [46]). Large bifacial cobble cores with flaking around the entire periphery were also a common byproduct of the experiments (Fig 10).

### Transitions between technological core types

Cores were classified into technological types after each flake was removed. Type definitions follow Moore et al. [66] and are based principally on the arrangement of flake removals. All cores produced within the first three blows were classified as ‘assayed objects’. After the fourth flake was removed, cores with one platform were classified as ‘single platform’ if the flakes were struck unifacially to one core face, and ‘bifacial’ if the flakes were struck bifacially to two core faces. Cores with two or more independent platform edges (i.e., the edges did not overlap) were classified as ‘multiplatform’. The independent platforms might be unifacial, bifacial, or a mixture of both (e.g., Fig 7).

The cobble experiments produced a different proportion of core types from the flake blank experiments (Table 12). As discussed previously, the morphology of flake blanks influenced the rapid establishment of a bifacial platform around the perimeter, resulting in a relatively greater proportion of bifacial ‘radial’ cores; conversely, the globular morphology of cobble cores influenced the development and persistence of independent platforms, resulting in greater proportions of multiplatform cores. Only 3.4% of the cobble reduction experiments failed to produce multiplatform cores, compared to 43.3% of the flake blank reduction experiments. Multiplatform cores on flake blanks were mostly produced (and disappeared) within the first 50% of blows, whereas most multiplatform cores in cobble reduction persisted throughout reduction.

**Table 12:** Technological core types produced in the experiments.

Core Type*	Cobbles		Flake blanks		All	
	N	%	N	%	N	%
Assayed object**	87	13.8	90	18.6	177	15.9
Bifacial radial core	274	43.5	349	72.0	623	55.9
Multiplatform Core	263	41.8	45	9.3	308	27.6
Single Platform Core	6	1.0	1	0.2	7	0.6
Total	630		485		1115	

*Core types after [66].

** Cores with more than one independent platform but three or less flake removals were classified as ‘assayed objects’ rather than ‘multiplatform cores’. Each experiment produced three assayed objects.

**Fig 11:**
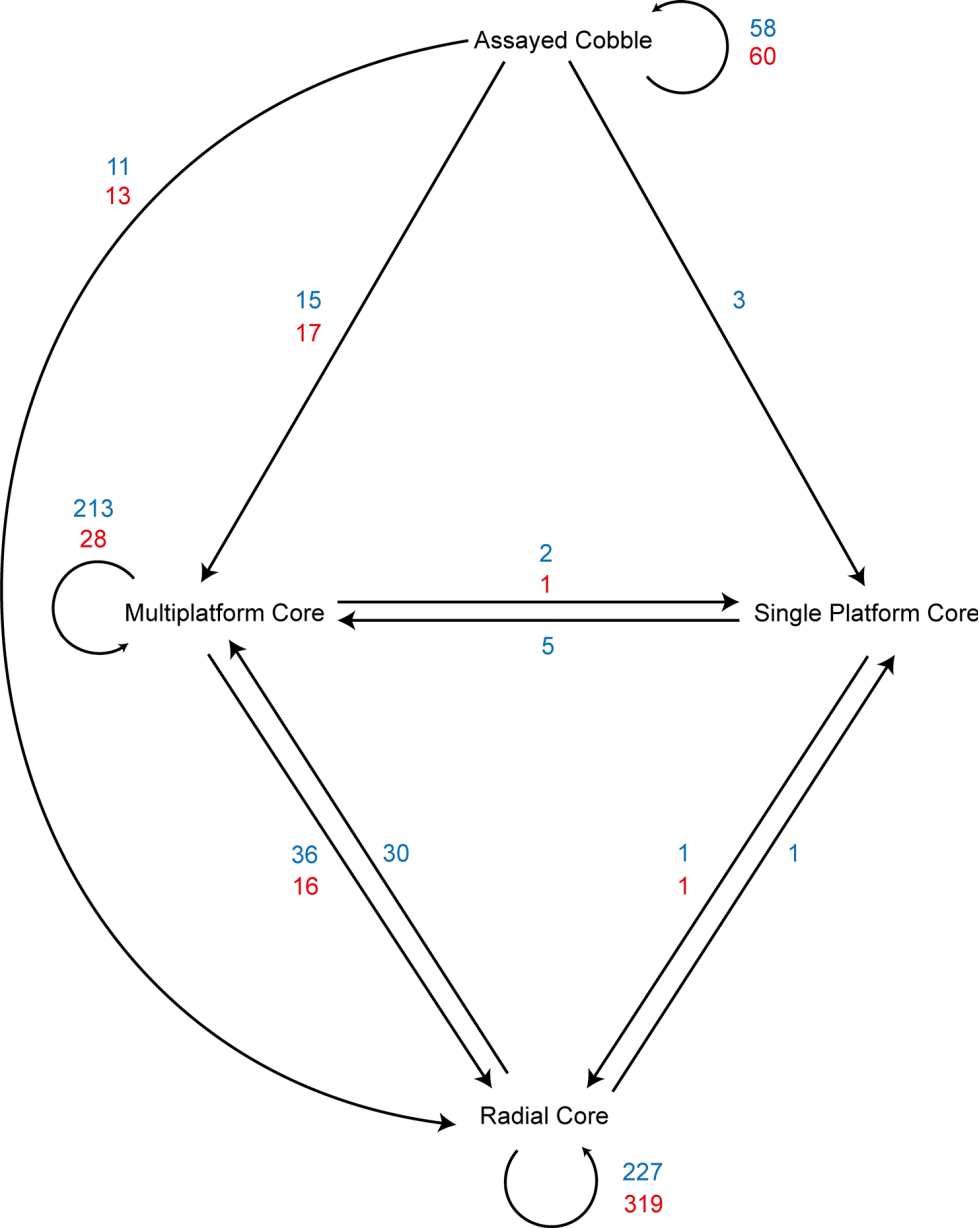
Frequency and direction of transitions between technological core types. Arrows indicate the direction of the transition, and numerals indicate the number of times a transition occurred in the experiments. Cores from the cobble experiments (N = 602) are in blue font, and cores from the flake blank experiments (N = 455) are in red font.

The cobble and flake blank experiments also differed in the proportions of transformations between technological core types (Fig 11). In general, flake blank reduction was less dynamic than cobble reduction. For example, once a core made on a flake blank transformed to a radial core, it never made the transition to a multiplatform core. This contrasts with cobble reduction, where 30 cores transitioned from radial to multiplatform. The starting morphology of the blanks was the key factor: the globular cobbles were characterised by more variation in possible platform/high mass configurations but, as discussed previously, flake blanks were characterised by clearly differentiated core volumes at the outset of reduction. This consistent starting configuration reduced the variation in platform/high mass configurations for the duration of flake blank reduction.

### Changes in core thickness through the reduction process

Relative changes in core thickness can be gauged by dividing maximum width by maximum thickness, referred to as the width-to-thickness (W:T) ratio [65]. In our experiments, the protocol of maximising flake size and randomising platform selection led to convergence on a similar W:T ratio for cores made on cobbles and flake blanks. At the outset of reduction, flake blanks were significantly thinner than cobbles, but the W:T ratios of cores were statistically indistinguishable at the end of reduction (Table 13). Flake blanks tended to decrease in W:T ratio from their starting point (becoming relatively ‘thicker’) and cobbles tended to increase in W:T ratio (becoming relatively ‘thinner’), converging on an average W:T ratio of about 1.8–1.9.

**Table 13:** Change in width/thickness from unmodified blank to final core.

Object type	Cobbles, Mean +/- SD; CoV, N = 29	Flake blanks, Mean +/- SD; CoV, N = 30	Unpaired *t* test result, cobbles vs. flake blanks
Unmodified blank	1.42 +/- 0.26; 18.6	2.64 +/- 0.61; 23.0	Significant (*p*<0.0001; *t* = 0.9030; SE = 0.122)
Final core	1.75 +/- 0.44; 24.9	1.86 +/- 0.46; 24.6	Not significant (*p* = 0.3584; *t* = 0.9260; SE = 0.116)

The physics of Hertzian cone formation in conchoidal fracture cause the interior platform angles on flakes to be a consistent 136 degrees regardless of variation in exterior platform angles [84]. This consistency is mirrored by negative scars, with a natural tendency for platform angles to homogenise as reduction proceeds, and this phenomenon is one of the ‘good tricks’ that modern knappers capitalise on to control the toolmaking process [42]. This sort of control was eliminated by the experimental design, so the results suggest that, once flakes begin to overlap at the centre of the core face, a W:T of about 1.8–1.9 may be the natural result of hard-hammer percussion delivered to non-margin platforms. Modern knappers resort to special platform preparation to overcome this effect and achieve ‘secondary thinning’ in bifacial reduction ([65]:35).

### Changes in core elongation through the reduction process

The elongation (L/W) of cores made on cobbles at the end of reduction was statistically indistinguishable from cores made on flake blanks (Table 14). Cobble elongation increased slightly during reduction, while flake blank elongation decreased slightly; the maximum elongation reached in cobble reduction was 2.00 and in flake blank reduction was 2.08. The final cores were, on average, 1.34 to 1.33 times longer than wide, and the similarity of these values may suggest a natural convergence similar to that seen for W:T ratios.

**Table 14:** Changes in length/width from unmodified blank to final core.

Object type	Cobbles, Mean +/- SD; CoV, N = 29	Flake blanks, Mean +/- SD; CoV, N = 30	Unpaired *t* test result, cobbles vs. flake blanks
Unmodified blank	1.29 +/- 0.18; 13.9	1.41 +/- 0.24; 17.2	Significant (*p* = 0.0461; *t* = 2.0393; SE = 0.059)
Final core	1.34 +/- 0.20; 14.8	1.33 +/- 0.24; 17.9	Not significant (*p* = 0.7329; *t* = 0.3430; SE = 0.057)

The core’s maximum dimension is length and width is the maximum dimension at right-angles to length. As a result of this convention, and because these dimensions were never precisely equal in the experimental datasets, all of the cores were slightly longer than wide.

It was previously proposed that elongation might occur by chance through greater attrition of core width than length ([42]:710), but this was not supported by these experiments. While cores sometimes became relatively elongated, this was always offset by removal of core ends, either through the selection of platforms in those positions or through tranchet-like end-lopping by the lateral expansion of flakes.

### Mass enhancement and core ‘predetermination’

**Fig 12:**
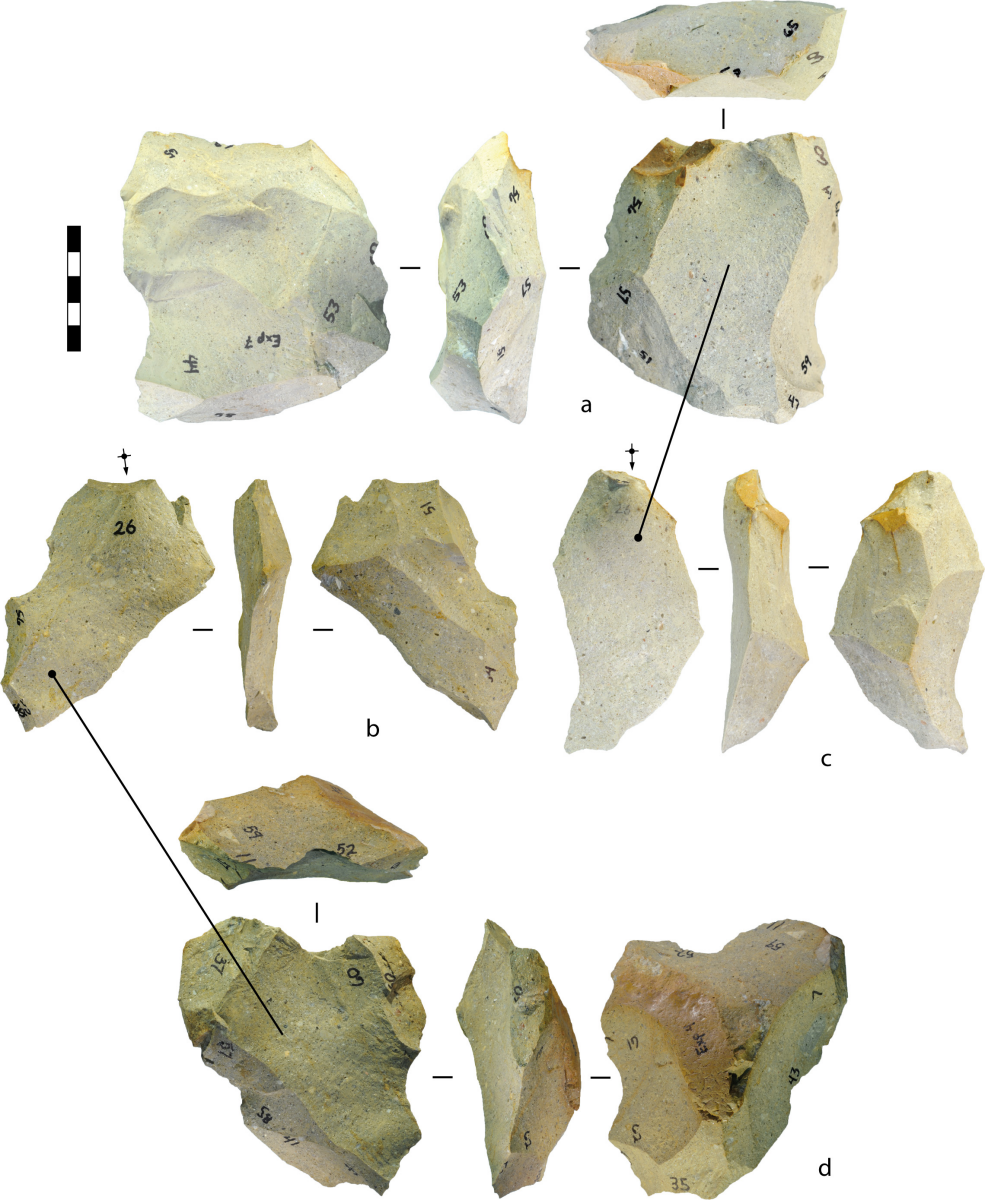
Experimental bifacial cores and ‘predetermined’ flakes. Both cores were made on silcrete cobbles. (A) Experiment 7, after blow 26. (B) Flake struck from the scar on D. (C) Flake struck from the scar on A. (D) Experiment 4, after blow 26. Scale 50 mm.

The Levallois Method [82, 85–87] involves strategic bifacial flaking to isolate core mass on one face, and then removing the mass with an invasive flake struck from a specially prepared platform. The Levallois flake—the intended outcome of the method—is removed roughly parallel to the plane of intersection of the two core volumes, as defined by the bifacial edge. The shape of this invasive flake is conditioned, or ‘predetermined’, by strategic flaking that enhances and shapes the core mass. Our experimental protocol of random platform selection precluded the strategic enhancement of core mass like that seen in the Levallois Method. Also, our protocols denied the knapper the option of manipulating platform angles by platform preparation. Nevertheless, core mass tended to become enhanced naturally by prior flaking, and in some cases suitably steep platforms were fortuitously situated to allow the invasive removal of ‘enhanced’ mass. In cases where such a platform was randomly selected, and the flake successfully removed, core forms were produced that possessed morphological attributes commonly attributed to deliberate ‘predetermination’ of the resulting flake (Fig 12).

This occurred 18 times in 11 of the 59 experiments, or 1.6% of the total number of blows (Table 15). The configuration for predetermination occurred 3.5 times more often in cobble reduction than in flake blank reduction, despite the lower odds that a given platform would be selected by the random number generator. Thicker cobbles were more prone to mass enhancement and steep platform configurations than thinner flake blanks. Given that the odds for selecting a particular platform were a relatively low 1 in 21.7 for cobbles and 1 in 15.2 for flake blanks, yet 1.6% of those selected platforms resulted in striking a ‘predetermined’ flake, the appropriate mass configurations must have arisen frequently during the experiments.

**Table 15:** Number and timing of ‘predetermined’ flake removals.

	Cobble	Flake Blank	All
No. (%) of predetermination configurations	14 (2.2%); N = 630	4 (0.8%); N = 485	18 (1.6%); N = 1115
No. (%) of experiments with predetermination configurations	7 (24%); N = 29	4 (13%); N = 30	11 (19%) N = 59
Earliest blow number, predetermination configuration	11	10	10
Mean and SD of reduction extent, as a percent of reduction by mass removed, when predetermination configuration created	87.4 +/- 6.5	93.3 +/- 2.6	88.7 +/- 6.3
Mean and SD of reduction extent, as a percent of reduction by blow count, when predetermination configuration created	79.0 +/- 14.8	85.1 +/- 15.2	80.4 +/- 14.6

A minimum of 10 blows preceded the configuration for predetermination, but this result is partly because, by convention (e.g., [82, 85, 87, 88]), the ‘predetermination’ configuration was recorded only when the relevant core mass was created by prior flake removals (and not by natural configurations on cobbles or pre-existing dorsal scar configurations on flake blanks). On average, this point was reached in the final 20% of core reduction, calculated relative to total blow counts and mass removal (Table 15). (Predetermined Levallois flakes were not produced until the final ~10% of cobble core reduction, as a proportion of initial starting mass, in knapping experiments by Texier ([73]:213). In our experiments, then, the configuration for predetermination was a function of reduction intensity. Three of the cobble experiments were carried over the weight threshold that ended the experiment by the removal of a ‘predetermined’ flake.

### Flake types

**Fig 13:**
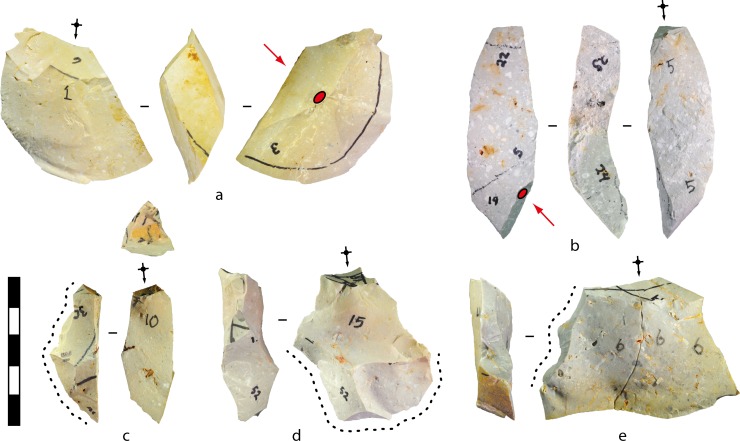
Flake types produced in the experiments. (A-B) Contact removal (‘Kombewa’) flakes produced in the flake blank experiments. The red arrows and dots mark the percussion axes and points of force application for detaching the flake blanks, and the black diacritical arrows show the platform location and percussion axes for the contact removal flakes. Flake B was struck burin-like down the edge of the flake blank. (A) Silcrete, experiment 4, blow 1. (B) Silcrete, experiment 25, blow 5. (C-E) Redirecting flakes produced in the flake blank experiments. The black diacritical arrows show the platform locations and percussion axes, and the dotted lines mark the part of the core platforms removed by the flakes. C was struck burin-like directly down the edge of the flake blank, D overstruck the end of the bifacial core, and E spread laterally in a tranchet-like fashion and removed part of the core’s margin. E was broken by a siret fracture. (C) Silcrete, experiment 12, blow 10. (D) Silcrete, experiment 3, blow 15. (E) Silcrete, experiment 27, blow 6. Scale 50 mm.

The flakes produced during the experiments were classified into three types (after [66]) (Fig 13). ‘Core-edge’ or ‘redirecting’ flakes preserve a former platform edge, with one or more negative point of force application (PFA) as a dorsal attribute, and were produced when the propagating flake undercut a pre-existing platform ([89]:22) (This definition does not refer to negative scars from prior reduction from the same platform. If present, these emanate from the flake’s dorsal platform edge.) In some cases the knapper deliberately targeted the high-mass platform edge, as discussed below, but in other cases a platform was undercut incidentally when a flake expanded laterally (e.g., a tranchet-like flake [90] or distally (e.g., an overstruck flake). A ‘contact removal’ flake (after [66])—a type of ‘Kombewa’ flake [91]—was produced when a flake undercut the bulb of percussion and positive PFA on a flake blank’s ventral surface. The positive PFA and all or part of the bulb of percussion are preserved as dorsal attributes on the contact removal flake. All other flakes were grouped into the ‘early reduction’ category.

The cobble and flake blank experiments produced these flake types in similar proportions (Table 16). Contact removal flakes could not be produced from cobbles, and when these are added to the early reduction flakes struck from flake blanks, the flake types struck from cobbles and flake blanks do not differ significantly (chi square = 0.377, df = 1, p = 0.54). Since the blank’s positive PFA must be present as a dorsal attribute to warrant classification as a contact removal flake, a maximum of one could be produced per experiment, and 91% of these were removed within the first five blows. However, only 22 of the 30 flake blank experiments produced a contact removal flake. In the other 8 experiments, the blanks’ positive PFAs were not clearly identifiable on the dorsal surfaces of the resulting flakes. All of the flake blank experiments produced multiple flakes with a dorsal ‘detachment scar’—a remnant ventral surface from the flake blank preserved on the dorsal surface of the flake [66].

**Table 16:** Flake types produced in the experiments.

	Cobbles	Flake Blanks
Early reduction	475 (77.5%)	355 (74.4%)
Redirecting	138 (22.5%)	100 (21.0%)
Contact removal	0	22 (4.6%)
Total	613	477

Flake type definitions after [66].

**Fig 14:**
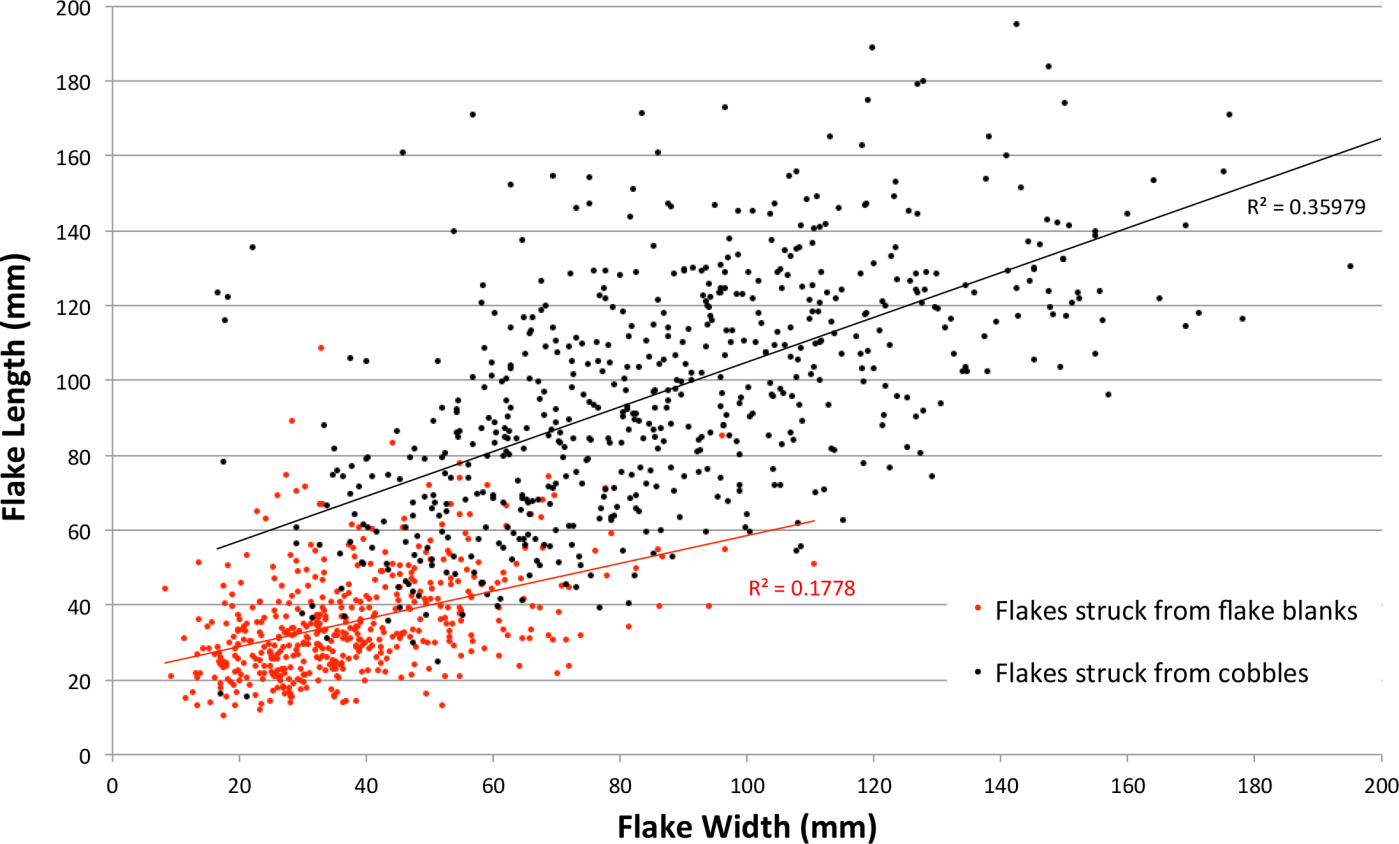
Scatterplot of flake lengths and widths produced in the cobble and flake blank reduction experiments.

The protocol guiding the experiments required the knapper to maximise flake mass; flake shape was not a consideration. Because of this, flake shape was not constrained by a deliberate focus on high mass areas of specific form, as occurs in blade-making. Nevertheless, zones of high mass were sometimes relatively long compared to their width, and flakes struck from mass of this shape were sufficiently elongated to be classified as ‘blades’ (Table 17). Average flake elongation was close to 1.0 for both sets of experimental flakes, and length and width are weakly correlated for both the cobble and flake blank reduction experiments (Fig 14).

**Table 17:** Elongation of flakes produced in the experiments.

	Flakes struck from cobbles, N = 604	Flakes struck from flake blanks, N = 471
Elongation (L/W), Mean +/- SD	1.21 +/- 0.62	1.05 +/- 0.54
Elongated flakes (L/W>2.0), N (%)	37 (6.1%)	32 (6.8%)

### Cortex coverage on flakes

**Fig 15:**
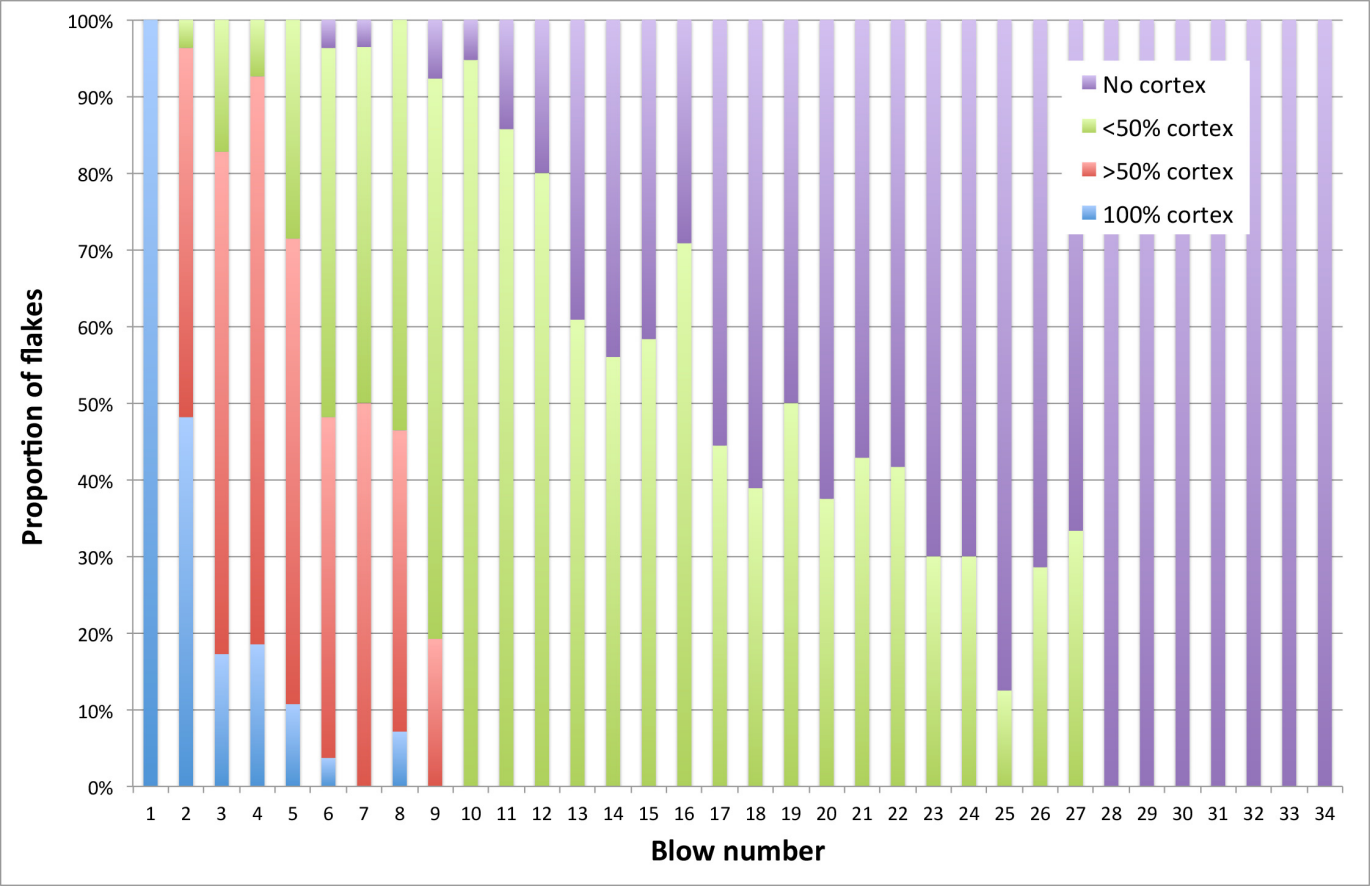
Flake cortex coverage by reduction interval (blow number). Sample sizes decrease towards the right-hand site of the chart because not all cores sustained the maximum 34 blows.

Changes in dorsal cortex coverage on flakes from cobble reduction are shown in Fig 15. Flakes with substantial amounts of dorsal cortex (>50%) disappeared within the first 9 blows, but small patches were often retained on cores to the end of a reduction experiment. Flakes entirely lacking cortex appeared as early as 6 blows but rapidly increased in proportion from about 10 blows. All cortex was eliminated from experimental cobbles cores that sustained 28 or more blows.

Toth [92] has suggested that the cortex distribution on flakes can reflect hominin handedness because of regularities in the biomechanics of core rotation when striking flakes unifacially from one platform edge. According to the model, right-handed knappers will create greater numbers of flakes with cortex on the right margin, and left-handed knappers will create greater numbers of flakes with cortex on the left margin ([92]:Fig 3). The knapper in our experiments was right-handed, and asymmetrical distribution of cortex of this sort was noted on 95 flakes, but the ratio of right-cortex and left-cortex flakes was nearly equal ([46] vs. [49]). This is because platforms were chosen randomly and therefore reduction moved randomly between the faces of a cobble, resulting in a very different core reduction process than the unidirectional reduction assumed by Toth’s model. The experimental pattern is closer to the theoretical distribution of right- and left-hand cortical flakes produced by ‘alternate flaking’, where flakes are struck sequentially to alternate core faces (cf. [47]).

### Burin scars and flake types

A burin scar is created when a flake propagates down an edge dividing two core volumes. In our experiments, the edges of flake blanks were an unambiguous division and served as a landmark for tracking burin scar production. Burin scars were created when the blank’s edge proved accessible from the sides of a prior negative scar, usually a platform in Position 1 or 2 (Table 18). In this case, the edge itself defined the centre of the zone of high mass targeted for flake removal, although sometimes the edge was located at the periphery of the targeted mass. If the edge served as a platform prior to this, a redirecting flake was produced, although the removal of other types of flakes also produced burin scars. The removal of most redirecting flakes—and nearly a quarter of contact removal flakes—created a burin scar on the core (Table 19).

**Table 18:** Platform positions for blows that produced burin scars on flake blank cores.

Position	N	%
1	66	50.8
2	14	10.8
3	15	11.5
4	35	26.9

**Table 19:** Flake types that produced burin scars on flake blank cores.

Flake Type	N	%
Contact removal (N = 22)	5	22.7
Early reduction (N = 355)	52	14.7
Redirecting (N = 100)	73	73.0

## Discussion

The earliest stone assemblages are characterized by ‘expedient’ ([49]:38), ‘simple’ ([47]:27), or ‘opportunistic’ ([20]:113) reduction by rote flaking [47, 49], although considerable perceptual-motor and cognitive skills were required to remove individual flakes [47, 93]. Core reduction was guided by an imprecise mental visualisation, and no concept of predetermining flake shape by prior flaking [94]. Pelegrin [47] identifies two reduction formulas, or ‘algorithms’, in these early assemblages: 1) adjacent flaking from a single platform surface which involved turning the core between removals (cf. [92]; e.g., [93]); and [2]) alternating bifacial flaking from a single platform edge which involved both flipping and turning the core between removals. The algorithmic nature of the flaking suggests that the morphology of the developing core was not monitored by the hominin stoneworker, even though core morphologies are distinctive and repetitive [47]. Raw material shapes strongly influenced approaches to core reduction and/or the shapes of most discarded cores [20, 47, 95]. Roche [49, 94] views bifacial and certain multiplatform flaking as intentional core shaping or ‘sculpting’, with the flakes as byproducts, and thus the cores reflect an early hominin ‘mental template’ ([49]:36, [94]). Bifacial reduction is a common element of these early hominin assemblages (e.g., [51, 53, 96–99]).

Our results show that bifacial reduction is inevitable when platforms are chosen randomly, and bifacial edges occurred on all cores within the first 12 blows. Bifacial cores converged on a W:T ratio of 1.8–1.9 and a L:W ratio of 1.33–1.34. The relatively low L:W ratio and centripetal scar patterning created centripetal or ‘radial’ cores. Blank shape influenced the shapes of cores produced early in our experiments (cf. [20]) but morphological and technological convergence occurred as reduction intensity increased. Although scars sometimes overlapped in a way that mimic’s Pelegrin’s ([47]:27–28) algorithmic alternate flaking, this was a chance result of random platform selection rather than an intention or flaking algorithm. Key features of the ‘discoidal reduction schema’ described by Boëda [82, 85] were produced in our experimental assemblage, including 1) two core faces reduced non-hierarchically relative to the plane of core face intersection (the ‘bifacial plane’); 2) creation and maintenance of peripheral convexities; and 3) reduction tangential to the bifacial plane, rather than parallel to it. Similarly, a proportion of the experimental cores were multiplatform.

**Fig 16:**
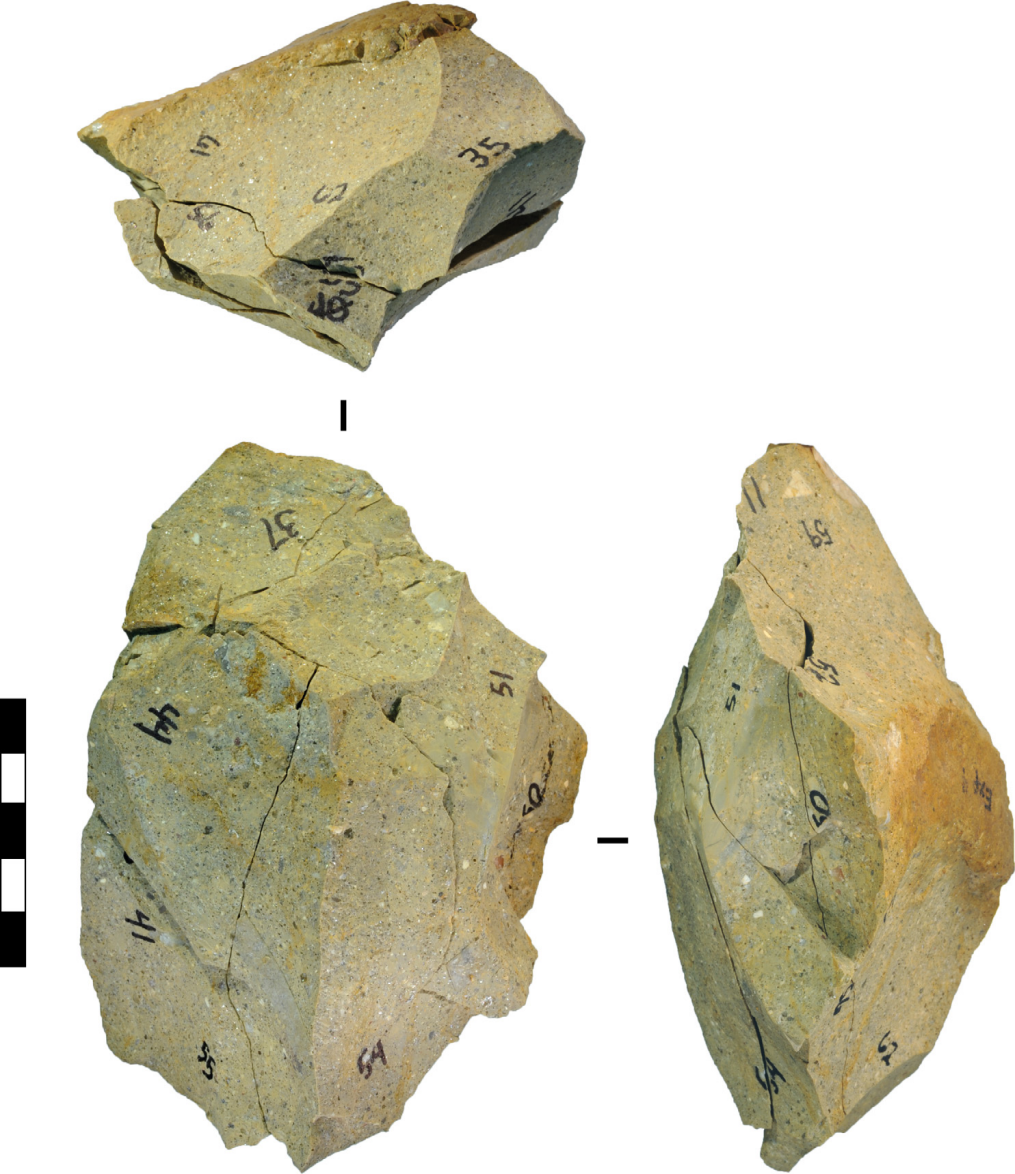
Experimental elongated bifacial core classifiable as a ‘handaxe’. The core was produced from reduction of a silcrete cobble, experiment 4, after 21 blows. Flakes were conjoined to reconstruct this core. Subsequent reduction produced the ‘predetermined’ flake shown in Fig 12B. Scale 50 mm.

**Fig 17:**
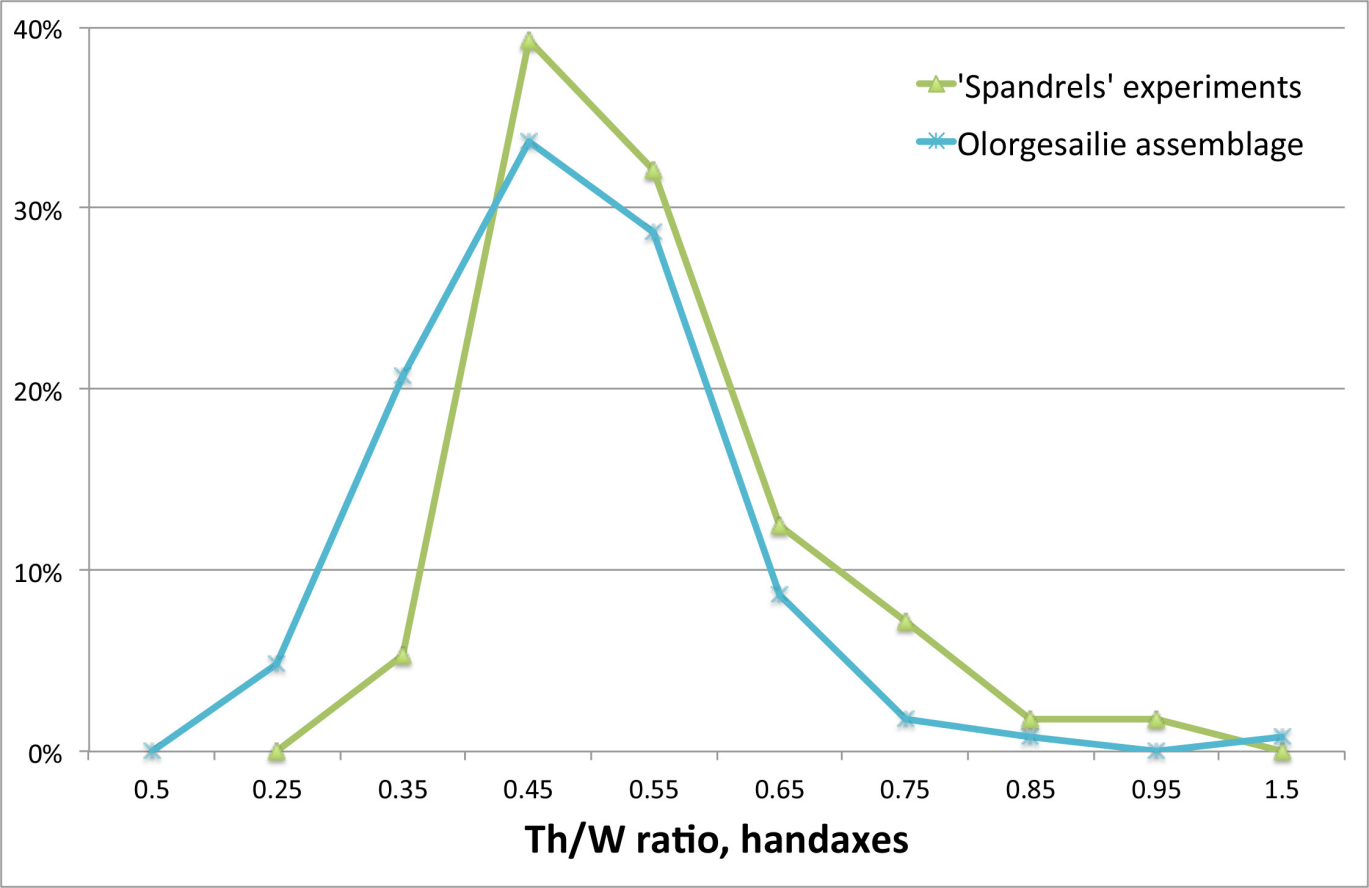
Proportion of Thickness/Width attributes for experimental handaxes and handaxes recorded by Isaac from Olorgesailie. Olorgesailie data from [67]:figure 41.

**Fig 18:**
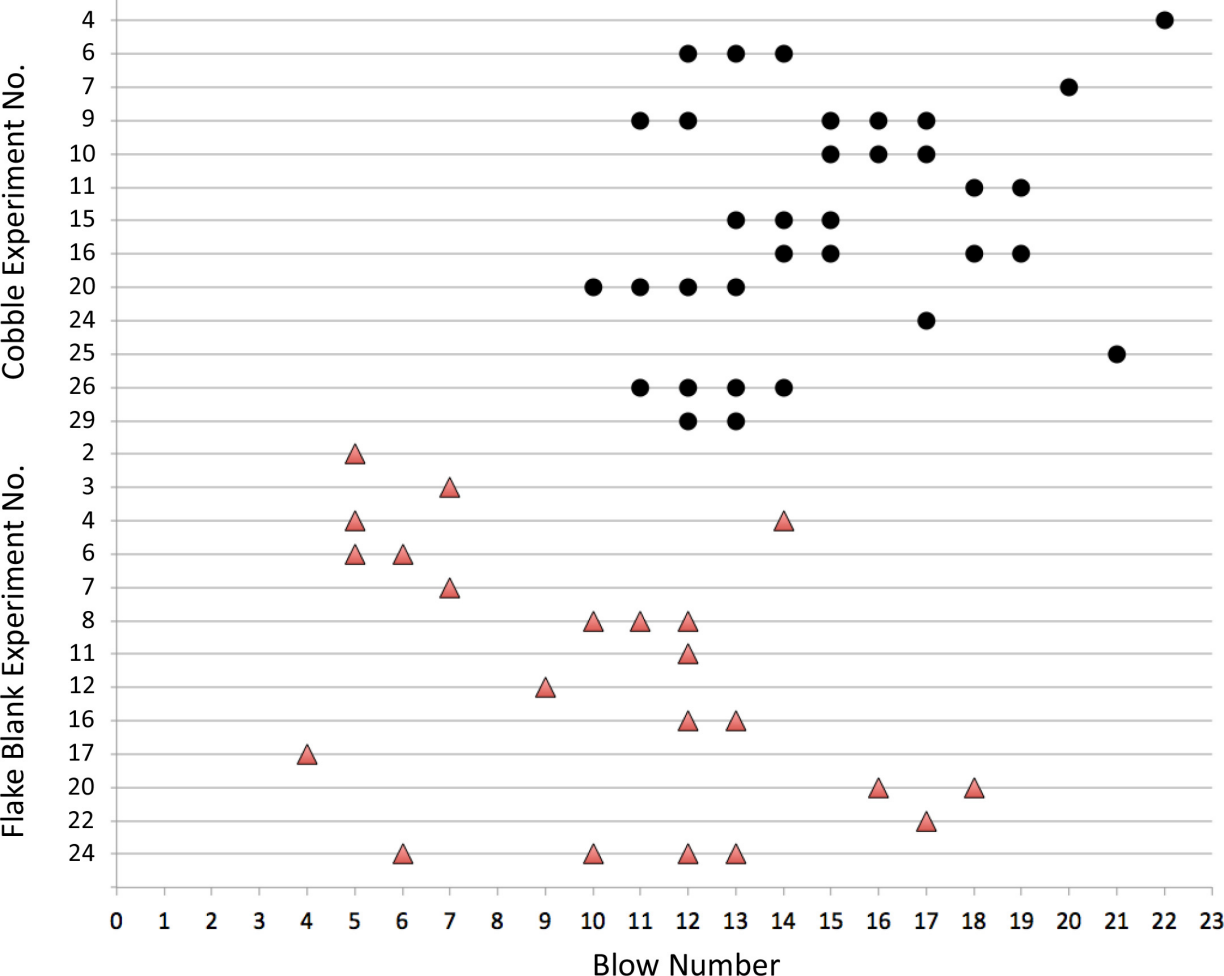
Production of experimental handaxes by reduction interval. Cores classifiable as handaxes (N = 56, see Table 20) occurred in 13 (43%) of the flake blank and 13 (45%) of the cobble reduction experiments. Handaxes occurred earlier in the flake blank experiments—probably influenced by flake blank starting morphology—but persisted for fewer numbers of sequential blows.

The bifacial handaxe first appears in the archaeological record about 1.75 mya. [100]. Most archaeologists view these elongated bifaces as intentionally produced core forms created according to a preconceived plan. Hominin knappers shaped handaxes relative to a bilateral plane and a bifacial plane, and the manufacture of symmetry in both planes simultaneously is seen as a significant cognitive breakthrough [8, 17]. The bifacial flaking, sharp durable cutting edge, circumferential working, broad symmetry, and ‘good prehensile qualities’ of bifacial handaxes is seen as evidence for ‘a mental construct’ ([101]:119–120, cf. [46]). Early handaxes or ‘proto-bifaces’ [96] ‘were unbalanced and crudely made’ because ‘the operational procedures were not yet mastered’ ([49]:42). Some 56 of the bifacial cores in our experiment were sufficiently elongated (Width/Length<0.76) and flattened (Thickness/Width<0.67) to be classified as ‘handaxes’ in Isaac’s [67] typological scheme for the Acheulean site of Olorgesailie (Table 20, Figs 16, 17 and 18). Nevertheless, they better resemble ‘proto-bifaces’ than the refined, symmetrical bifaces that emerged by the Late Acheulean, ca. 700 ka [57]. Late Acheulean handaxes were made using elaborated methods that involved progressive shifts in intermediate goals to achieve the ultimate goal of a ‘standardized product’ ([47]:27–28), a process that was made impossible by our strict experimental design.

**Table 20:** Comparison of attributes between experimental and Olorgesailie handaxes.

	Olorgesailie handaxes	Experimental handaxes		
	(Mean +/- SD)*	Cobble experiments (Mean +/- SD, N = 34)	Flake blank experiments (Mean +/- SD, N = 22)	Total (Mean +/- SD, N = 56)
Width/Length (Elongation) (W/L)	0.57 +/- 0.08	0.713 +/- 0.070	0.705 +/- 0.067	0.710 +/- 0.069
Thickness/Width (Th/W)	0.53 +/- 0.12	0.60 +/- 0.12	0.47 +/- 0.08	0.55 +/- 0.12
Maximum Length (mm)	165 +/- 13.9**	159.1 +/- 30.6	76.8 +/- 18.1	126.8 +/- 48.3

Including cores classified as handaxes, cleavers, pick-like handaxes, picks, and triedres. The attributes are those calculated by Isaac [67] for comparison to other early stone artefact assemblages.

*Isaac did not report measurements on all specimens, but the samples sizes are between 540–1023 ([67]:Table 10).

**Approximately 60 Olorgesailie handaxes measured less than 75 mm long, with the smallest measuring ca. 45 mm ([67]:Figs 35, 58).

The Levallois Method emerged ca. 250–500 ka in Africa [73] and ca. 300 ka in Europe [102]. According to Boëda’s [82, 85, 87, 103] volumetric definition of the Levallois Method, Levallois flakes—the intended product of the stoneworker—were struck from one face of bifacial cores while platform shaping flakes were removed from the opposite face: the core faces, or ‘volumes’, were arranged hierarchically. ‘Predetermination’ involved enhancing and shaping the mass on the principal core face by removing material from the lateral sides and distal end of the core. The Levallois flake was struck to remove this mass from a platform bevelled by flaking towards the secondary core face, and positioned at the proximal end of the core. The platform was oriented so that the Levallois flake propagated parallel to the plane defined by the bifacial edge, removing much of the core face [82, 85–87]. Wynn and Coolidge suggest that to successfully achieve a stoneworking plan of such complexity, the hominin must be capable of encoding the necessary technical knowledge and to enact this encoded information through a ‘retrieval structure’ triggered by physical cues on the developing core—key elements of ‘long-term working memory’ [43]. About 2% of our experimental bifacial cores reflect a Levallois-like pattern of reduction. The similar traits include 1) an invasive flake struck from a steeply angled platform and oriented parallel to the bifacial plane, and 2) production of core face convexities by prior flaking. The cores are dissimilar from Levallois *sensu stricto* [82, 85, 103] because the experimental design explicitly prohibited treating core volumes hierarchically and striking ‘predetermined’ flakes from prepared platforms. Nevertheless, the traits that did emerge in the experimental assemblage are among those highlighted in the Levallois Method *sensu stricto* as evidence for the necessity of long-term working memory [1, 43].

## Conclusion

Our experiments created a ‘least-effort’ [50] stone artefact assemblage by removing as large a flake as possible from a randomly-selected core platform. Reduction ‘intent’ was restricted to the perceptions and motor skills necessary to remove a single flake. Higher-order, goal-directed intentions to produce a specific tool or flake type were explicitly prevented by random platform selection. Thus, our experimental design explored the interplay of stoneworking constraints and random choices to see what products would result. Of course, hominin choices were not completely random, and for that reason it is unsurprising that our outcomes do not replicate all aspects of early hominin assemblages. Rather, our experiments are a first step towards understanding the minimal conditions necessary to regularly produce the forms we see in the early archaeological record.

**Fig 19:**
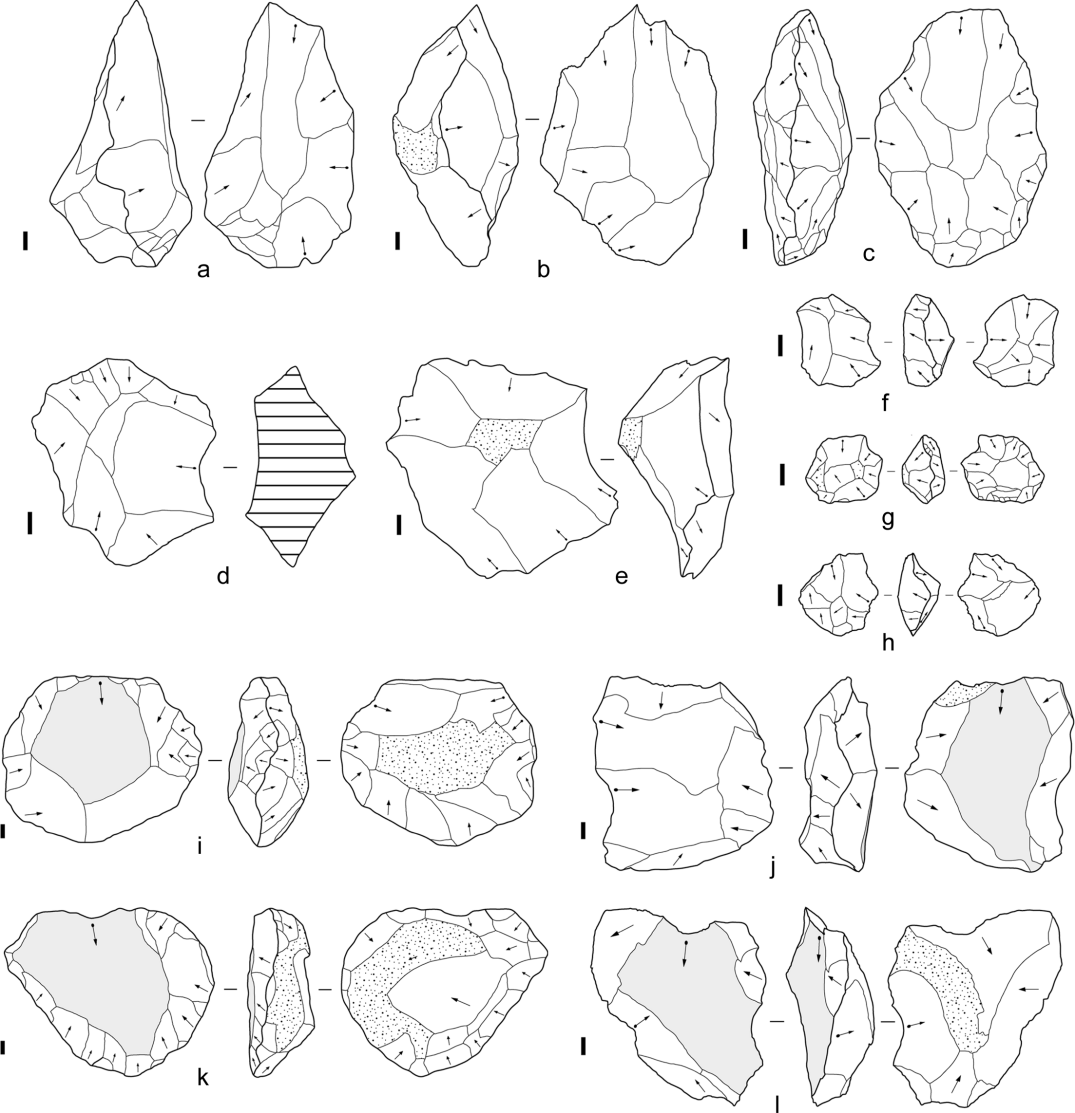
Experimental cores compared to archaeological specimens. (A) Volcanic, EF-HR, Olduvai Gorge ([96]:131). (B) Silcrete, cobble experiment 4, after blow 21. (C) Quartzite, Maropeng ([104]:9). (D) Volcanic, MFS, Olorgesailie ([67]:196). (E) Mudstone, cobble experiment 6, after blow 13. (F) Silcrete, flake blank experiment 10, after blow 19. (G) Chert, HWK East, Olduvai Gorge ([96]:105). (H) Silcrete, flake blank experiment 26, after blow 23. (I) Quartzite, Maropeng ([104]:10). (J) Silcrete, cobble experiment 7, after blow 27. (K) Quartzite DB3, Harts River ([52]:11). (L) Silcrete, cobble experiment 4, after blow 26. The ‘preferential scar’ is shaded on cores I-L. Scale bars 10 mm.

Despite our restrictive protocols, cores and flakes were produced that display aspects of the ostensibly ‘intentional’ flaking techniques and tool forms that mark early milestones in the standard story of technological and cognitive evolution (Fig 19). This included bifacial flaking that produced centripetally-organised cores, bifacial ‘choppers’ (cores with bifacial edges opposite cortical margins), cores reflecting a discoidal reduction schemata, and core attributes that mimic those produced by serial flake removal. Multiplatform cores similar to ‘polyhedrons’ were also produced. The shapes of cores changed in patterned ways through the reduction process, including the progression towards plateaus in biface width-to-thickness and length-to-width ratios (1.8–1.9 and 1.33–1.34 respectively). Certain elongated bifacial cores were classifiable morphologically as crude handaxes or ‘proto-bifaces’, and attributes suggesting ‘predetermined’ flake removals were created rarely but consistently. Blades and burins occurred, as well as burinated cores and tranchet-like tools. Although these stone artefact types and attributes mimic those often assumed to have been produced by goal-directed, intention-driven stone-flaking, in our experiments they were the outcome of the mechanics that govern stone fracture combined with a simple flake-removal algorithm applied repetitively to the same cores. Our results indicate that these types and attributes can be aspects of unintended design—stone-flaking ‘spandrels’ [42]—and, according to Wynn and Coolidge’s ‘standard of cognitive validity’ [1, 44], quoted at the outset of this paper, they might be considered outcomes of cognitively simple systems of flaking.

Studying assumed hominin design goals is now a mainstream approach to analysing early stone tool assemblages, as reflected in stone tool/hominin phylogenetics (e.g., [105–107]) and morphological taxonomy of artefacts (e.g., [14, 16, 108]); cognitive archaeology (e.g., [4, 17, 43, 46–49]), including brain-imaging studies (e.g., [30–35]); and cultural modelling of stone tools and hominin behaviour (e.g., [13, 109–114]). Results of studies like these inform general narratives about increases in hominin cognitive abilities in early human evolution (e.g., [2, 3]). However, our results complicate this picture because some of the patterning identified in these early assemblages may not reflect intentional ‘design’ in the usual sense.

Nevertheless, we did not produce iconic artefacts such as teardrop-shaped later Acheulean handaxes and Levallois Method cores *sensu stricto*, although the necessary conditions for these types emerged from the experiments. For handaxes, this included bifacial flaking, core elongation, and key core attribute ratios; and in the case of the Levallois Method, this included the enhancement of core mass and wholesale removal of this mass parallel to the bifacial plane (creating a ‘preferential’ flake). Given that, in our experiments, these patterns were an outgrowth of mechanical restrictions inherent to stone flaking, it seems likely that similar patterned byproducts were produced unintentionally by early hominins. Technological change may have involved hominin recognition of this patterning and enhancing the conditions that produced it. If so, the abundance of certain types and attributes in the archaeological record may be more significant than their first appearance, as this may track cognitive strategies to enhance particular stoneworking results. Developing the comparative ‘least-effort’ baseline of stoneworking byproducts [50] requires stoneworking experiments with protocols designed around design space restrictions rather than assumed hominin toolmaking intentions.

## Supporting Information

S1 Table Metric data, experimental cores and flakes. Attribute definitions are given in the text.
(XLS)

S1 Text Methodological influences on the experimental results. (DOCX)
