# Supplementary material for: Experimental Insights into the Cognitive Significance of Early Stone Tools
Source: PLoS One. 2016 Jul 8;11(7):e0158803. doi: 10.1371/journal.pone.0158803 (PMC4938430; doi:10.1371/journal.pone.0158803)
Supplement: S1 Text — (DOCX) [file pone.0158803.s002.docx]

**Methodological influences on the experimental results**

**Knapper idiosyncrasy and ‘black box’ gestures**

The knapping for these experiments was undertaken by one skilled flintknapper, raising the possibility that some of the documented effects were driven by his stoneworking knowledge and know-how ([1, 2], cf. [3]). The experimental design randomised the order of flake removal, effectively neutralising higher-order knowledge that might be prone to knapper idiosyncrasy, such as hierarchical strategic planning, recursive approaches to platform manipulation (e.g., [4]), and the ‘staging’ of flaking sets to achieve desired effects (e.g., [5]). Thus the possible idiosyncratic influence was restricted to the ‘black box’ perceptions and gestures necessary to remove a single flake (e.g., [6,-11]). Idiosyncrasies in gesture and perception sufficient to influence outcomes are less likely among skilled knappers than less-skilled knappers because the mechanics of fracture tightly constrain the range of technical options available to successfully apply the hard-hammer percussion technique. In these experiments, options were further restricted by formal application of the maximization protocol for each flake removed. Following the recommendation of Williams and Andrefsky ([12]), the experimental stoneworking was conducted by one flintknapper, eliminating the cross-subject effects apparent in studies of perceptual-motor stoneworking actions involved in removing single flakes (e.g., [6, 7, 9]). Nevertheless, ‘black box’ elements may have affected the experimental results in three ways.

First, knapper idiosyncrasy may have influenced the identification of suitable platform/mass relationships for flake removal, and, ultimately, the way that flake removals were chained together. For instance, the knapper frequently identified the edge of the developing core as an exploitable zone of high mass. Since core mass distribution is conditioned by prior flake removals, edge-targeting like this influenced the positions of platforms for subsequent reduction. Edge-targeting reflects the knapper’s experience in replicating cores associated with *Homo floresiensis* on Flores, Indonesia, where hominin knappers frequently struck flakes down edges of cores [13]. This may be an example where the knapper’s prior experience created a technical idiosyncrasy affecting the experimental results, particularly the production of burin flakes and scars.

Second, copper bars were used instead of stone hammers to maintain indentor consistency across all of the experiments and to aid in experimental replicability. This involved a trade-off in effectiveness because the malleability of the copper bars sometimes required the delivery of a greater number of blows to initiate fracture than might have been necessary with hard hammerstones (cf. [7]:54). This, in turn, may have influenced the unconscious ‘backing off’ in platform striking described previously, and therefore the accuracy of flake prediction, particularly relative to platform depth.

Third, a universal experience of knappers is that situation-specific issues can result in flake failure. These may include operational issues related to the nature of the raw material, mistakes in ‘black box’ gestures, as well as event-specific problems such as flaws in the stone ([2]:117). In our experiments, operational issues were controlled by using materials familiar to the knapper, although ‘black box’ miscalculations certainly occurred and factors such as stone flaws sometimes influenced the positions of potential platforms and zones of high mass on the core face.

Despite these issues, we believe that the sample size was large enough to ameliorate the influence of situation-specific factors. Knapper idiosyncrasy in mass/platform identification may have influenced core reduction trajectories, but, by using one knapper, the influence remained the same across all of the experiments. Although our major experimental findings are robust, future work with a variety of stoneworkers will be necessary to clarify the influence of knapper idiosyncrasy.

**Random platform selection and knapping rhythm**

Platform selection was randomized, forcing frequent core reorientations and disruption to free-flowing knapping ‘rhythm’. Random platform selection prevented the knapper from unconsciously influencing or directing flake removal patterns. In a sense, however, the strict adherence to random platform selection was more ‘designed’ than allowing the knapper to reduce the stone in a continuous knapping rhythm. For instance, human anatomy plays a strong role in the way that cores are rotated and manipulated by the non-dominant hand ([7, 10, 11, 14]), and hand-eye coordination might be improved through continuous gestural flow rather than isolated actions. Theoretically, these factors might allow for ‘intent-free’ flaking in adjacent multiple-flake sets struck from extended zones of feasible platforms, rather than the sort of constant and unpredictable core rotation forced by random platform selection—and the constant interruptions that were required to re-set the experiment for the next blow. Indeed, the fact that hominins undertook ‘much more knapping than was necessary’ ([15]:810) might suggest that hominins removed flakes in rhythmic gestural sets rather than focussing on the results of individual blows. Also, the maximization protocol may reflect greater design than simply removing ‘adequate’ flakes, and, as described above, this protocol demonstrably influenced the experimental results. These issues might be explored in future experiments that relax the random platform selection and flake maximization protocols, beginning with controlled and well-defined steps centred around the application of flaking ‘sets’ of pre-defined duration.

**References**

1. Pelegrin J. A framework for analysing prehistoric stone tool manufacture and a tentative application to some early stone industries. In: Berthelet A, Chavaillon J, editors. The use of tools by human and non-human primates. Oxford: Clarendon Press; 1993. pp. 302-314.

2. Pelegrin J. Prehistoric lithic technology–some aspects of research. Archaeol Rev Camb. 1990; 9(1): 116-125.

3. Moore MW. Bifacial flintknapping in the Northwest Kimberley, Western Australia. J Archaeol Method Theory. 2015; 22(3): 913-951.

4. Young DE, Bonnichsen R. Understanding stone tools: a cognitive approach. People of the Americas process series, vol. 1. Orono: Center for the Study of Early Man; 1984.

5. Callahan E. The basics of biface knapping in the eastern fluted point tradition: a manual for flintknappers and lithic analysts. Archaeol East N Am. 1979; 7:1-180.

6. Biryukova EV, Bril B, Dietrich G, Roby-Brami A, Kulikov MA, Molchanov PE. The organization of arm kinematic synergies: the case of stone-bead knapping in Khambhat. In: Roux V, Bril B, editors. Stone knapping: the necessary conditions for a uniquely hominin behaviour. Cambridge: McDonald Institute for Archaeological Research; 2005. pp. 73-89.

7. Williams EM, Gordon AD, Richmond BG. Biomechanical strategies for accuracy and force generation during stone tool production. J Hum Evol. 2014; 72: 52-63.

8. Ivanova GP. The biomechanics of the complex coordinated stroke. In: Roux V, Bril B, editors. Stone knapping: the necessary conditions for a uniquely hominin behaviour. Cambridge: McDonald Institute Monographs; 2005. pp. 119-128

9. Rein R, Bril B, Nonaka T. Coordination strategies used in stone knapping. Am J Phys Anthropol. 2013; 150: 539-550.

10. Williams EM, Gordon AD, Richmond BG. Upper limb kinematics and the role of the wrist during stone tool production. Am J Phys Anthropol. 2010; 143: 134-145.

11. Williams EM, Gordon AD, Richmond BG. Hand pressure distribution during Oldowan stone tool production. J Hum Evol. 2012; 62: 520-532.

12. Williams JP, Andrefsky Jr W. Debitage variability among multiple flint knappers. J Archaeol Sci. 2011; 38: 865-872.

13. Moore MW, Sutikna T, Jatmiko, Morwood M, Brumm A. Continuities in stone flaking technology at Liang Bua, Flores, Indonesia. J Hum Evol. 2009; 57:503-526.

14. Toth N. Archaeological evidence for preferential right-handedness in the Lower and Middle Pleistocene, and its possible implications. J Hum Evol. 1985; 14: 607-614.

15. Davidson I, William McGrew W. Stone tools and the uniqueness of human culture. J R Anthropol Inst. 2005; 11: 793-817.
